# IFN signaling is associated with radiotherapy response in malignant peripheral nerve sheath tumors

**DOI:** 10.1172/JCI195652

**Published:** 2026-03-02

**Authors:** Iowis Zhu, Julian Chien, Gabriel E. Rech, Kanish Mirchia, Sixuan Pan, Kaeli Miller, Joanna Pak, Rosanna Wustrack, Varun Monga, Steve E. Braunstein, Mark D. Adams, Line Jacques, Melike Pekmezci, S. John Liu, Harish N. Vasudevan

**Affiliations:** 1 Department of Radiation Oncology and; 2 Department of Neurological Surgery, UCSF, San Francisco, California, USA.; 3 The Jackson Laboratory for Genomic Medicine, Farmington, Connecticut, USA.; 4 Department of Pathology, Division of Neuropathology,; 5 Department of Bioengineering,; 6 Department of Orthopedic Surgery, and; 7 Department of Medicine, UCSF, San Francisco, California, USA.

**Keywords:** Genetics, Immunology, Oncology, Cancer, Cytokines, Radiation therapy

## Abstract

Patients with malignant peripheral nerve sheath tumors (MPNSTs) have poor outcomes despite multimodal treatment with surgery, radiation, and systemic therapy. The responses to radiotherapy (RT) are mixed, and the biologic mechanisms underlying this heterogeneity in the radiation response of MPNSTs are not understood. Here, we combined bulk and single-cell transcriptomics, genome-wide CRISPR interference screens, and multiplatform molecular analysis across MPNST cells, mouse allograft models, and patients’ samples to understand the mediators of the radiation response. Our data revealed that MPNSTs, but not benign plexiform neurofibromas, induced a type I IFN signature that functionally mediated the radiation response. Moreover, irradiation of immunocompetent mouse MPNST allografts led to IFN-mediated T cell recruitment and activation. Both host mouse T cells and intact tumor IFN receptor signaling were required for RT’s efficacy in mouse MPNST allografts. Analysis of human MPNST resection specimens demonstrated that increased microenvironmental and CD8^+^ T cell infiltration were associated with improved local control following RT. These results provide a preclinical rationale for combining immunomodulatory agents targeting IFN signaling to improve radiation responses in MPNSTs and potentially other soft tissue sarcomas.

## Abstract 2

This study elucidates transcriptional, functional genetic, and cellular mechanisms of interferon signaling that underlie radiotherapy response in people with MPNST.

## Introduction

Neurofibromatosis type 1 (NF-1) is an inherited condition affecting approximately 1 in 3,000 individuals worldwide (1). People with NF-1 are at increased risk for developing tumors of the PNS, including benign plexiform neurofibromas (pNFs) that can undergo transformation into malignant peripheral nerve sheath tumors (MPNSTs). MPNSTs are the most common cause of death in adults with NF-1 (2), and despite multimodal therapy with surgery, radiation and systemic therapy, outcomes remain poor for people with MPNSTs (3, 4), thus representing an urgent, unmet clinical need. In addition, people with NF-1 are at increased risk for secondary malignancies following radiotherapy (RT) (5), motivating an improved understanding of the therapeutic window for RT in this patient population.

MPNSTs are clinically managed as soft tissue sarcomas, a heterogeneous class of tumors treated with a combination of surgery, radiation, and systemic therapy tailored to patient- and tumor-specific factors (6, 7). Given the central role of RT in sarcoma management, prior groups have developed nomograms predictive for radiation response that integrate clinical and pathologic factors (8, 9), including specifically for MPNSTs (3, 10). However, these frameworks do not account for genetic mutations or microenvironmental composition, factors that can predict synergy with immunotherapy. Indeed, combining the immune checkpoint inhibitor pembrolizumab with preoperative radiation therapy improves outcomes when compared with RT alone for undifferentiated pleomorphic sarcomas or liposarcomas (11), underscoring the importance of the immune microenvironment in sarcoma treatment. However, whether this finding applies to other sarcoma subtypes, including MPNSTs, is not well understood. More broadly, the functional mediators and genetic basis for differential radiation response, as well as the mechanisms underlying the synergy between RT and immunomodulatory treatment approaches, remain unclear.

In this study, we sought to elucidate mechanisms underlying the radiation response in MPNSTs by combining bulk RNA-seq, genome-wide CRISPR interference (CRISPRi) screens, single-cell RNA-seq (scRNA-seq) of immunocompetent murine MPNST allograft models, and genomic analysis of human MPNST specimens. We found that MPNSTs were radioresistant and induced a distinct transcriptional signature compared with benign pNFs, as highlighted by enrichment for immunomodulatory pathways such as IFN signaling. Consistent with this observation, genome-wide CRISPRi screens converged on type I IFN signaling as a critical mediator of the radiation response. scRNA-seq of irradiated murine MPNSTs in vivo demonstrated that radiation increased T cell recruitment and activation with a concomitant induction of IFN secretion both by tumor cells and macrophages within the microenvironment. We also found that the ability of radiation to inhibit tumor growth was inhibited in both immunodeficient mice and when IFN signaling was knocked down in tumor cells. Finally, analysis of human MPNST specimens from patients treated with surgery and radiation revealed that the radiation response was associated with increased microenvironmental infiltration and CD8^+^ T cell presence and inversely associated with CDKN2A loss. Taken together, our study demonstrates the importance of IFN signaling from both tumor cells and the microenvironment in mediating radiation responses in MPNSTs, providing a preclinical rationale for immunomodulatory approaches to improve the efficacy of RT for sarcomas.

## Results

### MPNST cells demonstrate increased radioresistance and modulate IFN signaling pathways.

**Figure 1:**
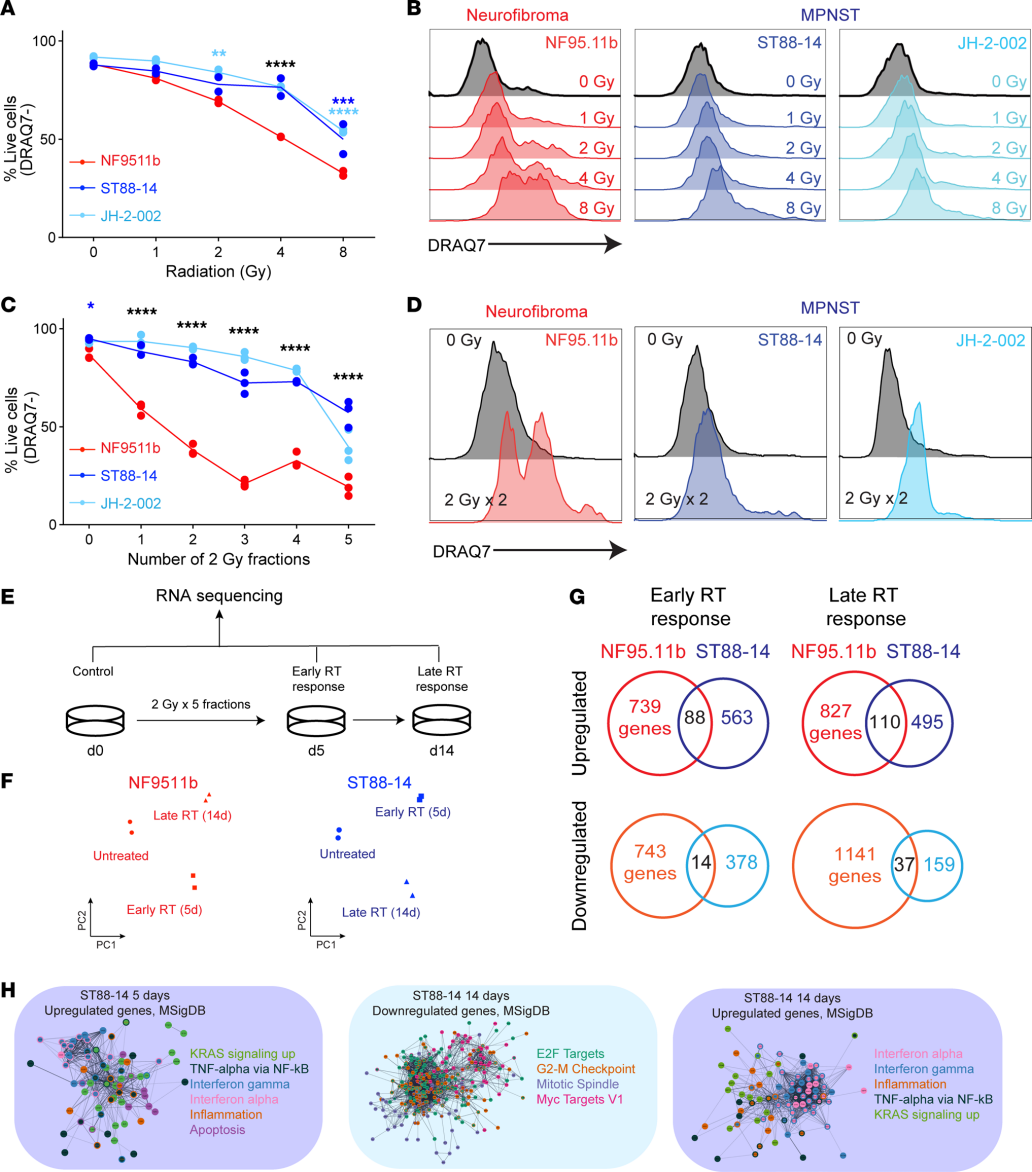
MPNST cells demonstrate increased radioresistance compared with pNF cells and transcriptionally modulate IFN signaling. (**A**) Percentage of live cells as measured by DRAQ7 staining for NF95.11b pNF cells compared with ST88-14 and JH-2-002 MPNST cells treated with single-dose irradiation on day 0 and harvested on day 4. (**B**) Representative histograms of DRAQ7 staining of the indicated cell lines at different radiation doses as compared with day 0. (**C**) Percentage of live cells as measured by DRAQ7 staining for pNF and MPNST cell lines treated with the indicated number of daily 2 Gy fractions and harvested 48 hours after completion of irradiation. (**D**) Representative histograms of DRAQ7 staining of the indicated cell lines after 2 fractions of 2 Gy. (**E**) NF95.11b pNF and ST88-14 MPNST cell lines were irradiated with 5 fractions of 2 Gy and harvested at 5 days (d5) and 14 days (d14) after irradiation for bulk RNA-seq. (**F**) Principal component analysis of bulk RNA-seq data reveals that radiation modulated the transcriptome in both NF95.11b pNF and ST88-14 MPNST cells. (**G**) Comparison of significantly upregulated and downregulated genes between cell lines and time points shows unique gene expression changes in NF95.11b pNF compared with ST88-14 MPNST cells. (**H**) GO analysis followed by STRING network and Cytoscape visualization of differentially regulated genes in ST88-14 MPNST cells during the early RT response (5 days) and the late RT response (14 days) reveals induction of IFN signaling and repression of cell-cycle progression. *P < 0.05, **P < 0.01, ***P < 0.001, and ****P < 0.0001, by Dunnett’s multiple-comparison test.

To measure the single-fraction radiation dose response in pNFs and MPNSTs, the 2 human MPNST cell lines ST88-14 (12) and JH-2-002 (13, 14) and the human pNF cell line NF9511.b (15) were treated with a single radiation dose ranging from 0–12 Gy. Cell survival was assessed using the viability dye DRAQ7 through flow cytometry and using colony-forming assays (Figure 1, A and B and Supplemental Figure 1A; supplemental material available online with this article; https://doi.org/10.1172/JCI195652DS1). MPNST cells demonstrated significantly increased viability at 4 Gy and 8 Gy compared with pNF cells. To measure survival following fractionated RT, cell lines were similarly treated with daily doses of 2 Gy for up to 5 days and stained with DRAQ7, which revealed that MPNST cells had significantly increased viability across multiple fractions (*P* < 0.0001, 2-way ANOVA) (Figure 1, C and D). Together, these data demonstrate the relative radioresistance of MPNST cells compared with pNF cells.

To identify post-RT transcriptomic changes between pNF and MPNST cell lines that could mediate the observed differences in RT responses, we next irradiated the pNF cell line NF9511.b and the MPNST cell line ST88-14 with 5 daily fractions of 2 Gy and collected cells at day 5 and day 14 after RT for bulk RNA-seq (Figure 1E and Supplemental Table 1). Principal component and differential gene expression analysis of RNA-seq data revealed that radiation globally modulated the transcriptome in both cell lines (Figure 1F and Supplemental Figure 1, B–E). We observed distinct sets of significantly upregulated and downregulated genes in response to radiation in pNF and MPNST cells (Figure 1G). Gene ontology (GO) analysis of these significantly regulated gene sets revealed that ST88-14 MPNST cells, but not NF9511.b pNF cells, significantly (*P* < 0.05, Fisher’s exact test, ENRICHR) upregulated genes related to IFN signaling during both the early and late RT response and downregulated mitosis-associated genes in response to RT (Figure 1H and Supplemental Figure 1F). In sum, MPNST cells demonstrated radioresistance compared with pNF cells and exhibited a distinct transcriptomic response to radiation that was enriched for IFN signaling transcriptional signatures.

### Genome-wide CRISPRi screen reveals contributors to the MPNST RT response.

**Figure 2:**
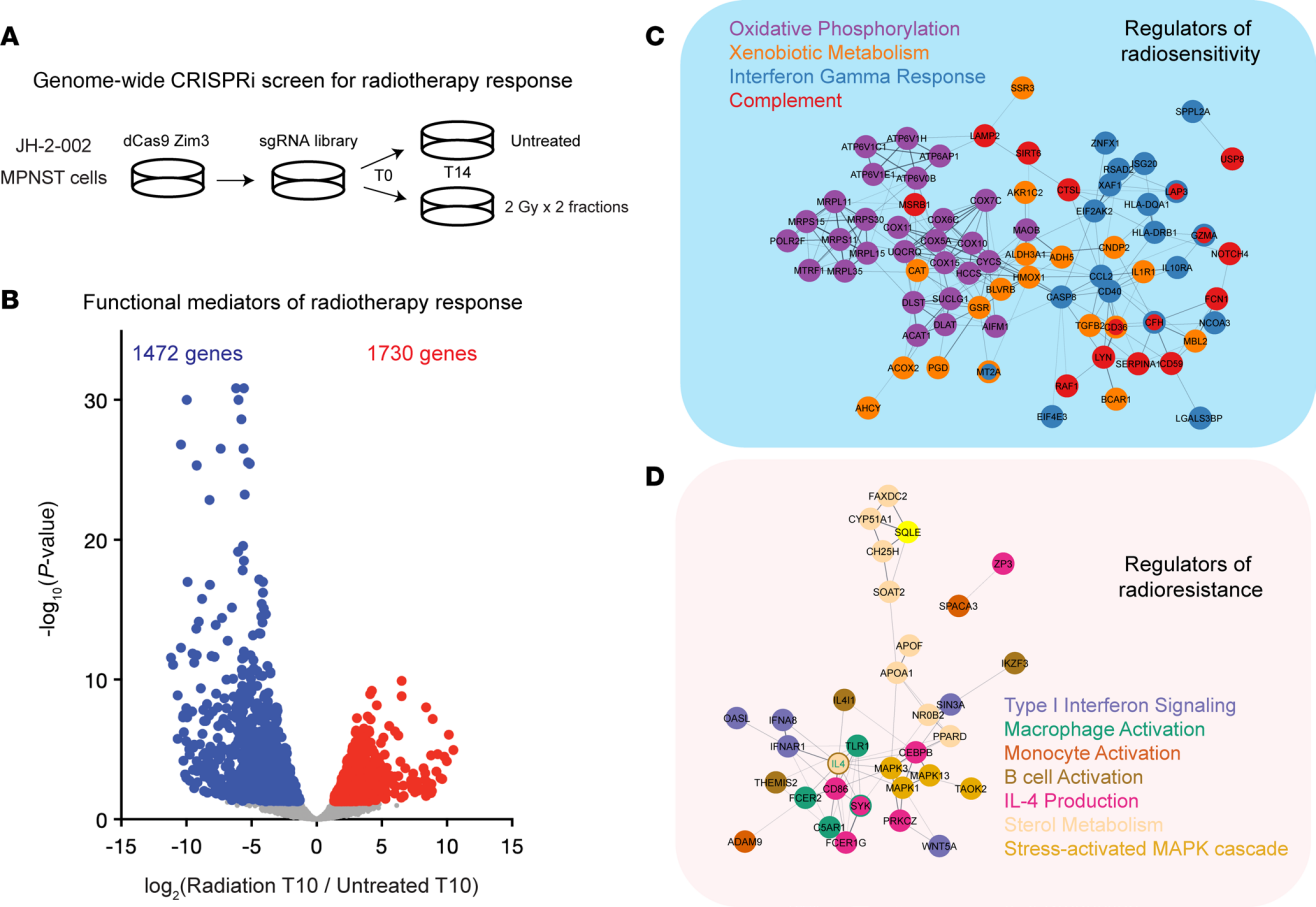
Genome-wide CRISPRi screen identifies functional mediators of the radiation response in MPNST cells. (**A**) Experimental setup of the CRISPRi screen in JH-2-002 MPNST cells. (**B**) Volcano plot depicting significantly enriched sgRNAs (*n* = 1,730, red) or depleted sgRNAs (*n* = 1,472, blue) between the irradiated and control groups (*P* < 0.05, Wald test). (**C**) GO analysis followed by STRING network visualization in Cytoscape of significantly depleted sgRNAs mediating radiosensitivity reveals enrichment for oxidative phosphorylation pathways (*NFE2L2*). (**D**) GO analysis followed by STRING network visualization in Cytoscape of significantly enriched sgRNAs mediating radioresistance reveals enrichment for IFN signaling as well as multiple cytokine and immunomodulatory pathways.

While our RNA-seq revealed changes in IFN gene signatures associated with the radiation response of MPNSTs, it remained unclear whether these differences were functionally required for radiation responses in MPNST cells. Thus, we next performed a genome-wide CRISPRi screen to identify functional mediators of the radiation response in JH-2-002 MPNST cells. To knock down individual genes in each cell, we transduced an established barcoded lentiviral dual single-guide RNA (sgRNA) library (16) into JH-2-002 human MPNST cells expressing dCas9-Zim3, with virus titrated to approximately 1 sgRNA vector per cell. Cells were sorted for library expression using FACS (Supplemental Figure 2A), and a T0 control was collected on day 0. Two experimental groups were designed, with 1 receiving 2 daily fractions of 2 Gy and the other being untreated, receiving no radiation. Genomic DNA (gDNA) from all cells was harvested on day 14, and sgRNA barcodes were amplified and sequenced (Figure 2A and Supplemental Table 2). To identify essential genes required for JH-2-002 MPNST cell growth, we first compared untreated T14 sgRNA abundances with T0 sgRNA abundances (untreated T14/T0), identifying 985 significantly enriched sgRNAs mediating increased cell growth and 323 significantly depleted sgRNAs mediating decreased cell growth (Supplemental Figure 2B). sgRNAs that inhibited cell growth were enriched for DNA repair pathways and chromatin remodeling (Supplemental Figure 2C) with noted genes *RREB1, PARP1*, and *KAT2B* (Supplemental Figure 2D). As expected, sg*TP53* repression significantly promoted cell growth (Supplemental Figure 2E).

**Figure 3:**
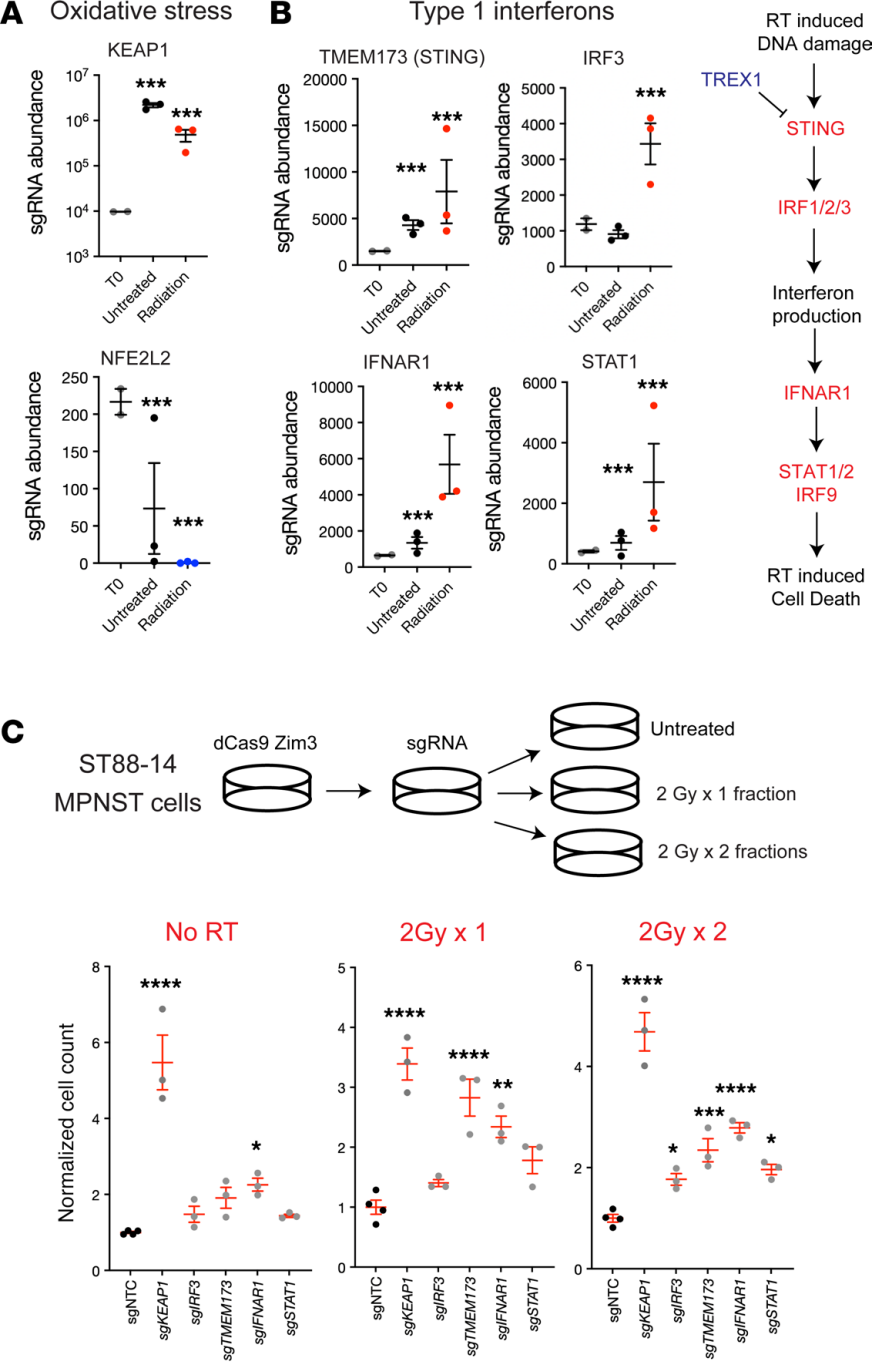
Type I IFN signaling suppression is sufficient for radioresistance in MPNST cells. (**A**) Normalized sgRNA abundances of *KEAP1* and *NFE2L2*, two members of the oxidative stress response (***P < 0.05, Wald test). (**B**) Normalized sgRNA abundances of members of the type I IFN signaling pathway: *TMEM173* (STING), *IRF3, IFNAR1*, and *STAT1* (***P < 0.05, Wald test). (**C**) Radiation responses in *sgKEAP1*, sg*IRF3*, sg*TMEM173*, sg*IFNAR1,* and sg*STAT1* validate these genes as mediators of the radiation response in human ST88-14 MPNST cell lines treated with 2 Gy for 1 or 2 fractions and harvested at 48 hours following the completion of irradiation. *P < 0.05, **P < 0.01, ***P < 0.001, and ****P < 0.0001, by Dunnett’s multiple-comparison test (**B** and **C**). Data are presented as the mean ± SEM.

A total of 1,730 sgRNAs were significantly increased in abundance after radiation (radiation T14/untreated T14), suggesting that these sgRNAs mediated radiation resistance, whereas 1,472 sgRNAs were significantly decreased in abundance, suggesting that these sgRNAs mediated radiation sensitivity (Figure 2B). GO analysis and Search Tool for Retrieval of Interacting Genes/Proteins (STRING) protein-protein interaction network modeling revealed that oxidative phosphorylation and stress sgRNAs were significantly enriched following radiation (Benjamini-Hochberg corrected *P* value < 0.05) (Figure 2C), consistent with the role of the Nrf2 signaling axis in modulating the radiation response (17, 18). sgRNAs significantly enriched in irradiated cells included components of the type I IFN response pathway (Benjamini-Hochberg corrected *P* value < 0.05) (Figure 2D and Supplemental Figure 3A). In addition, sgRNAs encoding previously reported mediators of radiosensitivity such as DNA repair genes (*ATM*, *RAD51AP1*) and TGF-β (*TGFB1*, *SMAD4*) were significantly depleted following RT (Supplemental Figure 3B), supporting the overall biological validity of our screen. In particular, *NFE2L2*, encoding the master oxidative stress response regulator NRF2, was significantly enriched among depleted sgRNAs mediating radiosensitivity, whereas *KEAP1*, a negative regulator of NRF2, and type I IFN response genes such as *TMEM173* (encoding the STING protein), *IRF3*, *IFNAR1*, and *STAT1*, were significantly enriched among sgRNAs mediating radiation resistance (Figure 3, A and B). (17, 18). We subsequently found that sg*KEAP1*, sg*IRF3*, sg*TMEM173*, sg*IFNAR1*, and sg*STAT1* ST88-14 MPNST cells had increased growth in response to 2 Gy × 1 fraction of radiation or 2 Gy × 2 fractions of radiation when compared with sgNTC ST88-14 MPNST cells treated with the same radiation dose (Figure 3C). However, it should be noted that all sgRNAs except sg*IRF3* also showed pro-growth effects in the no-RT condition. While this finding is consistent with the pro-growth effects of these sgRNAs in our CRISPRi screen (Figure 3, A and B), it may reflect multiple biological effects of these perturbations beyond modulation of the RT response alone. For experimental robustness, we further validated our findings in colony-forming assays across 2 MPNST models with single sgRNA CRISPRi repression of *KEAP1*, *IRF3*, or *IFNAR1* in both the JH-2-002 and human ST88-14 MPNST cell lines (Supplemental Figure 3, C–F). All 3 sgRNAs demonstrated significant target gene repression by quantitative reverse transcription PCR (qRT-PCR) in both JH-2-002 and ST88-14 cells (Supplemental Figure 3, C and D) with relative radioresistance observed in clonogenic assays (Supplemental Figure 3, E and F). Thus, our CRISPRi screen data identified multiple functional mechanisms underlying the radiation response in MPNSTs, including a key functional role for IFN signaling consistent with our bulk RNA-seq data.

### MPNST tumors demonstrate increased IFN and T cell signaling signatures in response to RT in vivo.

**Figure 4:**
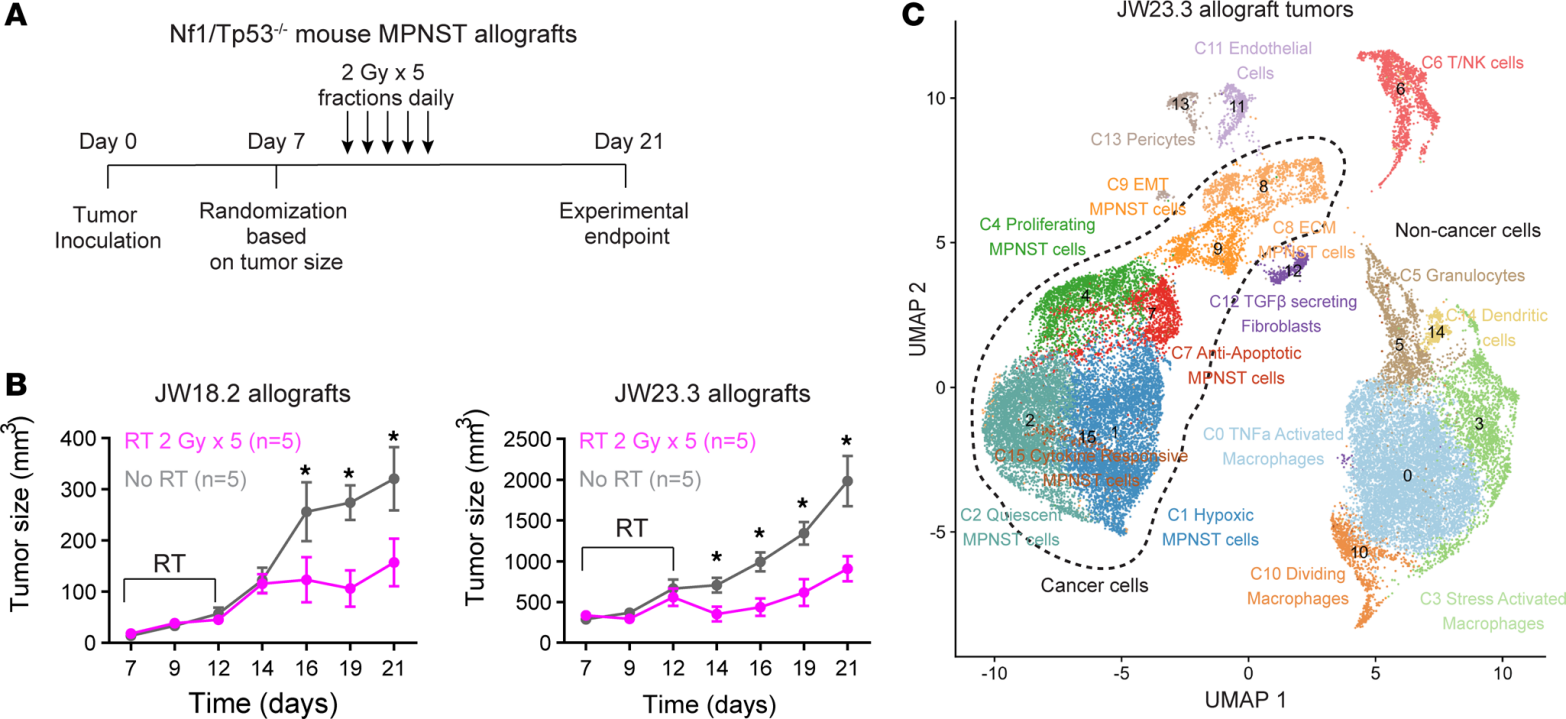
Irradiated mouse MPNST tumors contain multiple tumor and microenvironmental cellular subpopulations. (**A**) MPNST tumor irradiation in vivo experimental design with mouse *Nf1/Tp53*-mutant MPNST cells implanted subcutaneously in a C57/B6 WT mouse. (**B**) Growth curves of implanted JW18.2 and JW23.3 murine MPNST tumors reveal decreased tumor volume following irradiation (*P < 0.05, 2-sided Student’s *t* test). (**C**) Harmonized scRNA-seq UMAP of tumor and nontumor cell clustering of 32,763 cells harvested from JW23.3 tumors (*n =* 3 control, *n =* 4 irradiated) identified 7 tumor and 9 nontumor clusters.

**Figure 5:**
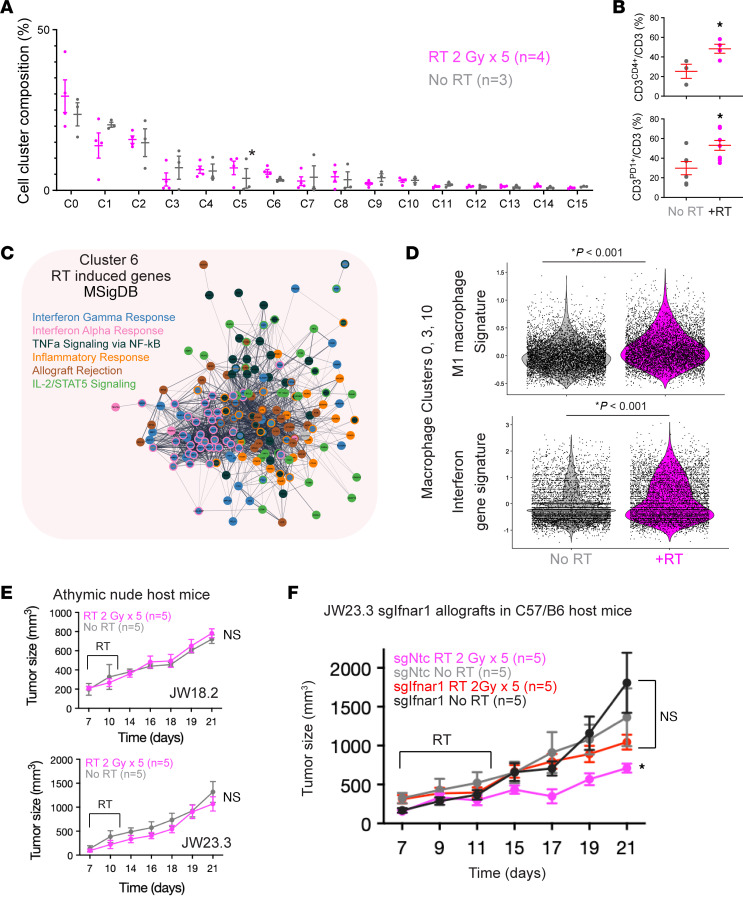
IFN activation and T cell recruitment are critical mediators of the MPNST RT response in vivo. (**A**). Breakdown of cell cluster composition by experimental groups reveals that only cluster 6 T/NK cells were significantly enriched in irradiated tumors (*P < 0.05, 2-sided Student’s *t* test). (**B**) Flow cytometric analysis showing the percentage of CD3^+^ cells that were also CD4^+^ between the experimental group (top) and the percentage of CD3^+^ cells that were also PD-1^+^ between experimental groups (bottom) revealed enrichment of both cell populations in irradiated tumors (*P < 0.05, 2-sided Student’s *t* test). (**C**) Differential gene expression analysis of irradiated versus control cluster 6 T cells and NK cells followed by GO biological process and Cytoscape visualization of STRING network of RT-induced genes in cluster 6 reveals upregulation of T cell receptor–based (TCR-based) T cell activation (**D**) Increased M1 macrophage polarization as shown by violin plots of IFN gene signatures and M1 macrophage gene signatures. (**E**) Radiation responses in JW18.2 or JW23.3 MPNST subcutaneous allografts implanted into athymic nude mice revealed that T cells were required for the radiation response. (**F**) JW23.3 sg*Ifnar1-*deficient MPNST cells did not respond to radiation therapy. *P < 0.05 and ***P < 0.001 versus the sgNTC no-RT control, by Dunnett’s multiple-comparison test.

Given that IFN signaling is critical for mediating crosstalk between the tumor and immune microenvironment (19), we next sought to better understand MPNST radiation responses in an immunocompetent in vivo model. We thus subcutaneously implanted WT C57/B6 mice with 2 different *Nf1/Tp53* double-KO murine MPNST cell lines, JW18.2 and JW23.3 (20–22), and randomized them into 2 experimental groups: 5 daily treatments of 2 Gy versus untreated tumors (Figure 4A). Tumors receiving RT had significantly reduced growth (Figure 4B). To define the transcriptomic changes and cellular subpopulations mediating the radiation response in these immunocompetent MPNST models, we next performed scRNA-seq on tumors from mice implanted with JW23.3 MPNST cells treated with radiation (*n =* 4) versus untreated (*n =* 3) tumors at day 21. Uniform manifold approximation and projection (UMAP) analysis revealed 7 tumor clusters and 9 nontumor clusters (Figure 4C, Supplemental Figure 4, A and B, and Supplemental Table 3), which were defined using cluster marker genes (Supplemental Figure 4C, Supplemental Tables 4 and 5), automated cell-type classification (Supplemental Figure 4D) (23), *Xist* expression restricted to host female mice rather than male tumor cells (Supplemental Figure 4E), and cell-cycle phase assignment (Supplemental Figure 4F). Of these clusters, only cluster 6, corresponding to T and NK cells, with expression of *PTPRC*, *CD4*, and *CD8* (Supplemental Figure 5A), was found to be significantly increased in irradiated tumors compared with unirradiated control tumors (Figure 5A). Flow cytometric analysis of the CD3^+^ T cell subset in an independent experiment was notable for a significant increase in CD4^+^ T cells and PD-1 expression following RT (Figure 5B), confirming the changes observed in our scRNA-seq data. A marked trend toward increased PD-1 expression in the CD3^+^/CD4^–^ compartment (presumed CD8 compartment) was also noted in irradiated versus nonirradiated tumors (Supplemental Figure 5B). Differential gene expression analysis of irradiated cluster 6 T and NK cells compared with unirradiated control cluster 6 T and NK cells revealed enrichment of genes important for TCR signaling and T cell activation such as *Gzmb* and *Zap70*, as well as type I IFN signaling genes (Figure 5C and Supplemental Figure 5C). We next performed differential gene expression analysis across the macrophage cell clusters 0, 3, and 10 between irradiated and control tumors (Supplemental Figure 5, D and E). GO analysis revealed a significant upregulation of type I and II IFN response pathways as well as a significant shift toward M1 macrophage polarization in irradiated macrophages as compared with control macrophages (Figure 5D and Supplemental Figure 5F). Finally, we performed differential expression analysis across MPNST cell clusters to determine tumor cell–autonomous mechanisms of the radiation response. GO analysis of tumor cell clusters 1 and 4 was notable for significantly upregulated expression of NFE2L2 target genes, suggesting a mechanism of tumor cell–intrinsic radioresistance through an NRF2-mediated oxidative stress response, consistent with our CRISPRi screen data (Supplemental Figure 6, A and B). Finally, irradiated cluster 7 tumor cells were enriched for genes encoding IL-2 and the type I IFN response pathway, supporting the importance of tumor cell autonomous IFN signaling in mediating RT responses as observed in our bulk RNA-seq and CRISPRi screen data (Supplemental Figure 6C). No significant differentially regulated gene sets between irradiated and control cells were observed in the remaining tumor cell clusters. To determine whether host mouse T cells are required for radiation efficacy, we next implanted JW18.2 or JW23.3 mouse MPNST cells into athymic nude mice, which did not respond to RT (Figure 5E). Finally, we implanted sg*Ifnar1*-deficient JW23.3 mouse MPNST tumor cells into C57/B6 mice, which showed a significantly dampened response to RT *(P* < 0.05) as compared with mice implanted with sgNTC control cells (Figure 5F). In sum, both tumor-intrinsic and microenvironmental type I IFN secretion underlying T cell recruitment are induced following RT and appear to be required for radiation efficacy in mouse MPNST allograft models.

### MPNST post-RT clinical outcomes are affected by tumor purity and CD8^+^ immune cell infiltration.

**Figure 6:**
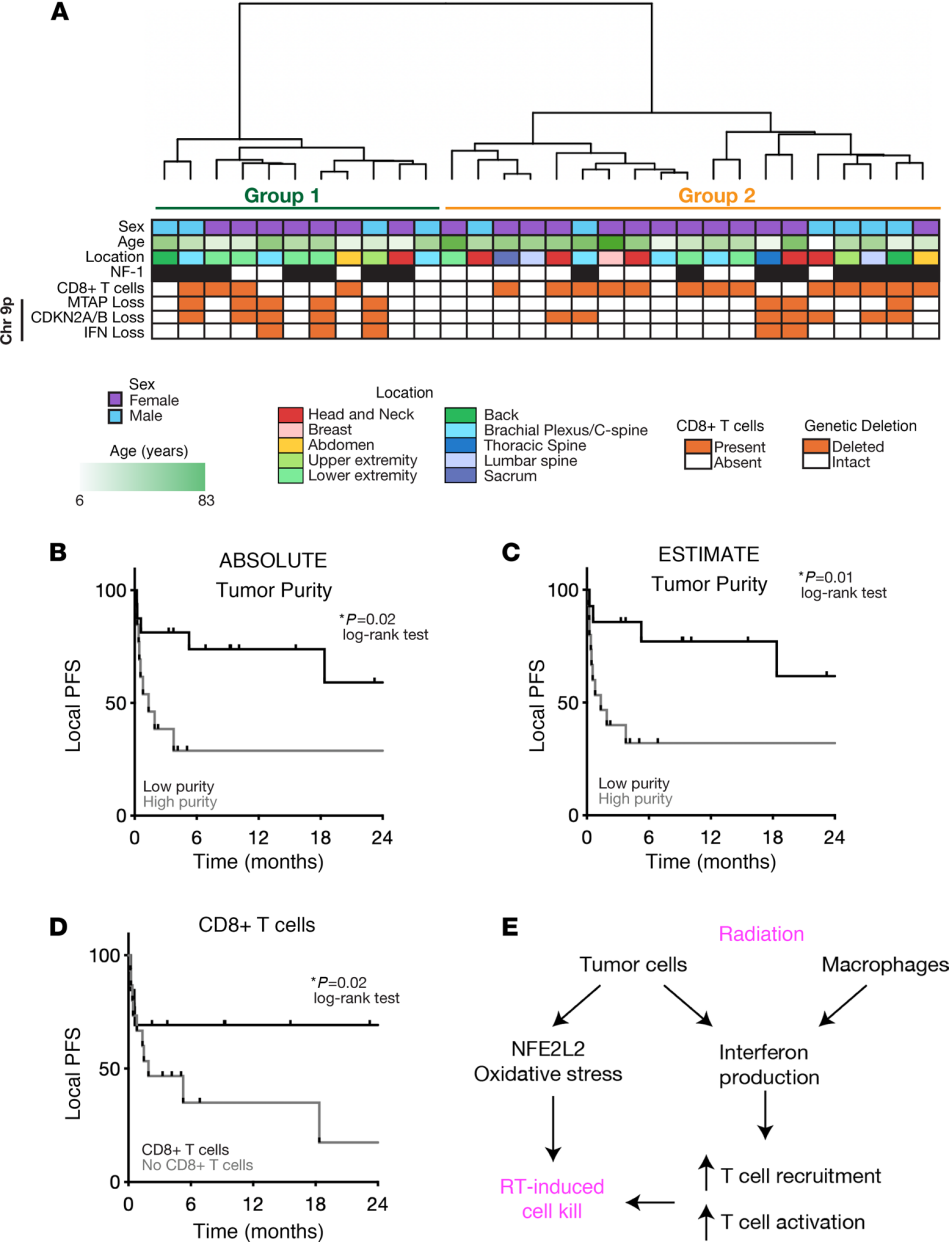
Microenvironmental composition is predictive of the RT response in a human MPNST cohort. (**A**) Unsupervised hierarchical clustering of DNA methylation arrays from resected human MPNST specimens (*n =* 30) revealed 2 clusters. Chr, chromosome. (**B** and **C**) Decreased tumor purity reflecting increased microenvironmental composition as measured by (**B**) ABSOLUTE or (**C**) ESTIMATE is associated with improved local PFS following RT. (**D**) Methylation cell-type deconvolution–based estimate of CD8^+^ T cells shows that the presence of CD8^+^ T cells is associated with improved local PFS following RT. (**E**) Model summarizing the tumor cell–autonomous and microenvironmental effects of radiation on MPNSTs converging on IFN production underlying T cell recruitment to drive radiation-induced cell killing. *P* values in **B**–**D** were determined by log-rank test.

While our in vitro and in vivo analyses support the importance of IFN signaling and T cell recruitment as critical for radiation responses in MPNSTs, the relevance of these putative predictive biomarkers for people with MPNSTs is not clear. We previously identified immunohistochemical patterns associated with the RT response in people with MPNSTs (3), but the predictive utility of microenvironmental composition is not well understood. We thus assembled an updated cohort of 30 patients with MPNSTs treated with surgical resection and postoperative radiation, who underwent methylation profiling with detailed clinical follow-up. Unsupervised hierarchical clustering identified 2 epigenetic groups (Figure 6A and Supplemental Figure 7, A and B). Epigenetic group 1 tumors had significantly increased copy number variants (CNVs) but no significant difference in age, NF-1 status, or biologic sex (Supplemental Figure 7, C–F). Immunomethylomic deconvolution (24, 25) revealed that group 1 tumors harbored significantly increased CD4^+^ T cells and significantly decreased monocytes, with a trend toward decreased CD8^+^ T cells compared with group 2 tumors, suggesting that microenvironmental composition was associated with epigenetic group (Supplemental Figure 7G). Next, we sought to determine whether microenvironmental composition was associated with the RT response as measured by local progression-free survival (PFS). Tumor purity estimation using either the ABSOLUTE or the ESTIMATE method (26) demonstrated that low-purity tumors with increased microenvironmental signatures were associated with improved local PFS, suggestive of an improved RT response (Figure 6, B and C). Similarly, MPNST stratification by CD8^+^ T cell infiltration (present versus absent) revealed that patients with tumors harboring CD8^+^ T cells had significantly improved local PFS (Figure 6D). Finally, the only recurrent genetic alteration that was significantly associated with local PFS in this cohort of patients was *CDKN2A/B* loss with chromosome 9p21.3 (Supplemental Figure 9A and Supplemental Table 6). In addition to *CDKN2A/*B, which has been previously implicated in the malignant transformation of MPNSTs, the chromosome 9p21.3 locus (Supplemental Figure 8) contains both the *MTAP* gene and the IFN gene cluster. Analysis of *MTAP* or *IFN* locus deletion revealed trends toward poorer PFS, although these associations were not statistically significant (Supplemental Figure 9, B and C). Similarly, while all patients with deletion of the *IFN* locus had an absence of CD8^+^ T cells, this difference was not statistically significant, most likely because of our limited sample size (Supplemental Figure 9D). Taken together, our human MPNST cohort reinforces the importance of the immune microenvironment and T cell recruitment potentially underlying radiation responses in patients with MPNSTs (Figure 6E), consistent with our functional genomic and scRNA-seq analysis.

## Discussion

MPNSTs are aggressive PNS tumors that are the most common cause of death for individuals with NF-1. Despite multimodal therapy, clinical outcomes for patients with MPNST remain poor, indicating an unmet need for improved treatment strategies. Here, we combined bulk RNA-seq, genome-wide CRISPRi screens, and scRNA-seq of immunocompetent mouse MPNST allografts with molecular analysis of patients’ MPNST specimens to identify a critical role for IFN signaling and the immune microenvironment in modulating radiation responses (Figure 4E).

The differential radiation response between NF-1–associated neurofibromas (NFs) and MPNSTs has important clinical implications, given the concern for secondary radiation–induced malignancies in people with NF (5). Indeed, our data suggest that MPNSTs maintained both a survival and proliferation advantage over pNFs in response to RT and exhibited a distinct antiapoptotic and antiinflammatory transcriptional signature across both early and late time points compared with pNFs in vitro. Radioresistance has been previously described in both benign (27, 28) and malignant PNS tumors (29), suggesting mechanisms of action independent of a tumor’s mitotic or invasive ability. While the basis for these differential RT responses remains to be fully elucidated and validated, one explanation is that *NF1^–/–^* pNFs that lack the additional co-alterations present in MPNSTs, such as chromosome 9p loss or PRC2 mutation, are intrinsically more sensitive to RT, while these same alterations in MPNSTs support a stronger prosurvival response to RT. However, given the microenvironmental changes observed upon transition from pNFS to MPNSTS (30, 31), it is also possible that non–tumor cell–autonomous mechanisms such as IFN signaling may contribute to both transformation and RT responses within MPNSTs.

MPNST and pNF cells are known to display globally different transcriptional profiles (22, 32, 33). In line with this, we observed that pNF and MPNST cells expressed different levels of IFN-related genes at baseline. However, we did not observe uniformly higher or lower basal type I IFN signaling between MPNST and pNF cell lines at baseline. In addition, we found that genes that were significantly upregulated in MPNST cells at post-RT day 14 were found to be expressed at similar (IRF7, LGALS3BP), higher (GBP4), and lower (BST2, HERC6, IFI44L, IFIT3, IRF9) levels at baseline as compared with pNF cells (Supplemental Figure 1F), suggesting that the dynamics of differential type I IFN signaling could not be fully explained without a contribution from radiation.

Radiation-induced type I IFN signaling, whether from tumor cells or the immune microenvironment, was observed across multiple MPNST models. Prior studies have demonstrated a mixed relationship between IFN signaling and tumorigenesis, with both pro-oncologic (34) and tumor-suppressive roles (35) described in the literature. Clinically, IFN signaling through STAT1 has been shown to be critical for RT responses (36–39). These observations have led to clinical trials combining radiation with IFNs that have shown some efficacy but substantial toxicity (19), underscoring the narrow therapeutic window for systemic IFN modulation. One major roadblock to therapeutically leveraging IFNs in cancer has been the complexity of IFN signaling, both with respect to upstream inputs regulating IFN target gene expression and downstream outputs mediating the effects of IFN activation (40). By combining bulk RNA-seq, genome-wide CRISPRi screens, and scRNA-seq with a clinically annotated MPNST cohort, our study builds on the connection between IFNs and the radiation response by defining specific IFN signaling nodes (*TMEM173*, *IFNAR1*), transcription factors (*IRF3*, *STAT1*), and gene sets required for a RT response in MPNSTs. Indeed, although our CRISPRi screen, by nature of the required high cell density conditions, did not recapitulate the growth conditions for clonogenic growth, we observed the promotion of MPNST radioresistance following knockdown of multiple type I IFN–related genes in our validation studies. From a tumor heterogeneity perspective, we observed type I IFN modulation in both tumor cells and microenvironmental macrophages with associated macrophage polarization toward an M1 state (Figure 3G), leading to the post-RT recruitment of T cell populations in vivo. Interestingly, we observed that the benefits of radiation in inhibiting tumor growth were eliminated with either loss of the host immune system or tumor cell IFN signaling. Combined, these data underscore the importance of multiple IFN and chemokine sources arising from both the tumor and the microenvironment in determining the antitumor immune response. In contrast, our CRISPRi screen data suggest that knockdown of type II IFN signaling occurred to promote radiosensitivity, indicating a potential radioprotective effect of type II IFNs that has been observed in recent work (41). Additional validation followed by mechanistic interrogation to determine why some IFN signaling nodes, but not others, mediate RT responses will be an important area of future investigations to identify both diagnostic biomarkers and therapeutic approaches that would synergize with RT.

Analysis of our clinical cohort suggests that increased microenvironmental infiltration is associated with improved radiation responses, whereas chromosome 9p loss is associated with a decreased radiation response and T cell infiltration. We also observed differences in the proportion of CD4^+^ T cells between methylation clusters and an association between the presence of CD8^+^ T cells with the radiation response, consistent with previous literature linking radiation-induced type I IFNs to CD8^+^ T cell function (42). It is important to consider that methylation-based deconvolution is limited in its ability to define distinct immune subpopulations, and orthogonal validation by more robust methods such as flow cytometry, which require that tumor samples be collected prospectively following radiation, will be an important next step to confirm our methylation-based signatures and better delineate the immune cell subpopulations that mediate differences in microenvironmental composition. Moreover, given the rarity of MPNSTs and other sarcoma subtypes, observations within our single-institution cohort will no doubt require future multi-institutional, ideally prospective, studies.

Chromosome 9p deletion with an emphasis on *CDKN2A/B* loss is a well-established event in malignant transformation from pNFs to MPNSTs (43). However, the effect of additional genes beyond *CDKN2A/B*, such as *MTAP* and the type I IFN gene cluster, that are lost within the chromosome 9p21.3 region remains unclear. *MTAP* deletion leads to selective PRMT5 inhibitor sensitivity (44, 45), which shows therapeutic efficacy in preclinical MPNST models (46) and could be combined with RT. We also speculate that chromosome 9p–mediated IFN locus deletion may provide a genetic basis for impairing IFN activation and the radiation response in MPNSTs. Indeed, chromosome 9p21 loss is associated with immunotherapy resistance across multiple cancers (47, 48), and mouse genetic studies further suggest this IFN codeletion underlies immune evasion (49). However, the relationship between 9p loss, with its concomitant IFN codeletion, and RT responses remains an area in need of further investigation with larger patient cohorts. In addition, it will be equally critical to unravel the specific ligand-receptor pairs mediating the crosstalk between MPNST cells and the microenvironment following RT as well as the specific T cell subpopulations regulated by these signals. Finally, many other genes on chromosome 9p21, including *FOCAD* (50), may form the basis for additional synthetic lethal therapeutic combinations targeting this recurrent alteration.

In sum, the present work suggests that multiple mechanisms converge on type I IFN signaling to drive immunomodulation of T cell populations underlying the RT response in MPNSTs. Future work more precisely defining the mechanisms, regulators, and effectors of type I IFNs will be critical to design better-tolerated therapies that leverage these immunologic circuits, perhaps through direct STING agonists (51). In addition, given the limited number of available MPNST samples from patients who had undergone adjuvant RT, our clinical analysis is underpowered, and further multi-institutional efforts will be important to determine if these observations hold up in larger cohorts. Finally, our data establish the preclinical rationale for testing combination approaches with RT designed to stimulate IFN secretion, which can perhaps be best achieved with intralesional approaches (52), such as through intratumoral oncolytic virus administration (53), as a rational approach to improve radiation responses for these aggressive tumors.

## Methods

### Sex as a biological variable.

Our study examined male and female human samples, and similar findings are reported for both sexes. Mouse MPNST allografts were implanted exclusively into female mice in accordance with institutional practices, and we anticipate that the findings will be applicable to male host mice.

### Tissue culture.

Patient-derived NF (NF95.11b) or human MPNST (JH002-2, ST88-14) cell lines were obtained from the Neurofibromatosis Therapeutic Acceleration Program or the American Type Culture Collection (ATCC). Mouse MPNST cell lines (JW18.2, JW23.3) were a gift from Angie Hirbe (Washington University, St. Louis, Missouri, USA). Cell lines were grown in DMEM (11960069, Life Technologies, Thermo Fisher Scientific) with 10% FBS and 1× penicillin-streptomycin (15140122, Life Technologies, Thermo Fisher Scientific). Cell lines were regularly tested and verified to be mycoplasma negative (LT07-218, Lonza). For clonogenic assays, 100–1,000 NF or MPNST cells were seeded in 6-well plates, irradiated at the indicate dose/fractionation, and then grown for 7–14 days. Cells were fixed in methanol for 30 minutes and stained with 0.01% crystal violet (C6158, MilliporeSigma) for 1 hour. Plates were rinsed with water 3 times, allowed to air dry, and digitally scanned.

### Flow cytometry.

For proliferation assays, cells were stained with CellTrace Yellow (Thermo Fisher Scientific) according to the manufacturer’s protocol. Cells were then treated with a single fraction of radiation using an X-Rad 320 irradiator (Precision X-ray) or no radiation and incubated for 96 hours after completion of the radiation. For survival assays, cells were irradiated with a daily course of 2 Gy fractions and stained with DRAQ7 (Thermo Fisher Scientific) 96 hours after irradiation. Flow cytometry and FACS were performed on a BD FACSAria Fusion and analyzed using FlowJo software. Samples of cell suspensions from each tumor sample were stained with phycoerythrin-conjugated (PE-conjugated) anti–mouse CD45 (catalog 50-149-85, clone HI30, APC, Invitrogen, Thermo Fisher Scientific), PECy7-conjugated anti–mouse CD3e (catalog 560591, 17A2, BD Biosciences), BUV-conjugated anti–mouse CD4 (catalog 565974, GK1.5, BD Biosciences), and BV786-conjugated anti–mouse PD-1 (catalog 568565, 29F.1A12, BD Biosciences). Flow cytometry was performed as described above.

### Nucleic acid extraction and qRT-PCR.

RNA was extracted from the cell lines using the RNeasy Mini Kit (74106, QIAGEN) according to the manufacturer’s instructions, and cDNA was synthesized using the iScript cDNA Synthesis kit (1708891, Bio-Rad). Real-time qPCR was performed using PowerUp SYBR Green Master Mix (A25918, Thermo Fisher Scientific) on a QuantStudio 6 Flex Real Time PCR system (Life Technologies, Thermo Fisher Scientific) and analyzed using the double delta method as previously reported (22). The following qPCR primers were used: *GAPDH* forward (5′-GTCTCCTCTGACTTCAACAGCG-3′), *GAPDH* reverse (5′-ACCACCCTGTTGCTGTAGCCAA-3′); *KEAP1* forward (5′-CGGGGACGCAGTGATGTATG-3′), *KEAP1* reverse (5′-TGTGTAGCTGAAGGTTCGGTTA-3′); *IRF3* forward (5′-GAGAGCCGAACGAGGTTCAG-3′), *IRF3* reverse (5′-CTTCCAGGTTGACACGTCCG-3′); *TMEM173* forward (5′-CCAGAGCACACTCTCCGGTA-3′), *TMEM173* reverse (5′-CGCATTTGGGAGGGAGTAGTA-3′); *IFNAR1* forward (5′-AACAGGAGCGATGAGTCTGTC-3′), *IFNAR1* reverse (5′-TGCGAAATGGTGTAAATGAGTCA-3′); *STAT1* forward (5′-CAGCTTGACTCAAAATTCCTGGA-3′), and *STAT1* reverse (5′-TGAAGATTACGCTTGCTTTTCCT-3′).

### Bulk RNA-seq.

Library preparation was performed using the TruSeq RNA Library Prep Kit v2 (RS-122- 2001, Illumina). Single-end reads (50 bp) were sequenced on an Illumina HiSeq 2500 or a NovaSeq to a minimum depth of 25 million reads per sample at Medgenome Inc. Following quality control of FASTQ files with FASTQC, trimming of adapter sequences, reads were filtered to remove bases that did not have an average quality score of 20 within a sliding window across 4 bases (http://www.bioinformatics.babraham.ac.uk/projects/fastqc/) was performed prior to mapping. Reads were mapped to the appropriate reference genome (hg19) using HISAT2 with default parameters (54). Transcript abundance estimation in transcripts per million (TPM) and differential expression analysis were performed using DESeq2 (55). Principal component analysis was performed in R using the prcomp function. Differentially expressed transcripts with an adjusted *P* value of less than 0.1 were identified and filtered on the basis of an expression cutoff (TPM >1) and a fold change (FC) threshold (log_2_ FC >1) to prioritize biologically relevant gene sets. Significantly enriched gene ontologies were selected using ENRICHR (56) and visualized using the stringApp package in Cytoscape.

### CRISPRi cell line generation and genome-wide screening.

Lentivirus containing UCOE-SFFV-dCas9-BFP-ZIM3 (188777, Addgene) was produced from transfected HEK293T cells with packaging vectors (pMD2.G 12259, Addgene, and pCMV-dR8.91, Trono Laboratory) following the manufacturer’s protocol (MIR6605, Mirus). JH-2-002 cells were stably transduced to generate parental JH-2-002^dCas9-Zim3-BFP^ cells and selected by flow cytometry using a SH800 sorter (Sony). Subsequent gene-specific knockdowns were achieved by individually cloning sgRNA protospacer sequences into the pCRISPRia-v2 vector (84832, Addgene) between BstXI and BlpI restriction sites. All constructs were validated by Sanger sequencing of the protospacer region. The following protospacers were used: sgNTC (GTGCACCCGGCTAGGACCGG); sgKEAP1-1 (GGCCCTGGCCTCAGGCGGTA); sgKEAP1-2 (GTGGAGCCGAGGCCCCCCGA); sgIRF3-1 (GGGAGGCGTCTACACTGAGG); sgIRF3-2 (GGGGTGGACTCCGTAGATGG); sgTMEM173-1 (GAGAGCAGCCAGTGTCCGGG); sgTMEM173-2 (GGGTGCCCAGCCACTCCCAG); sgIFNAR1-1 (GTAACTGGTGGGATCTGCGG); sgIFNAR1-2 (GATGTAACTGGTGGGATCTG); sgSTAT1-1 (GGCAGGAAAGCGAAACTACC); and sgSTAT1-2 (GCTGCGCAGAGTCTGCGGAG). Lentivirus was generated as described above, and cells were selected to purity using 1–5 μg/mL puromycin for at least 5 days.

For genome-wide CRISPRi screening, we used a compact and highly active barcoded sgRNA library that was optimized through aggregation of 126 genome-wide CRISPRi screens, established sgRNAs targeting essential genes, and machine-learning prediction algorithms (22). This genome-wide dual sgRNA library has been previously validated through multiple growth-based screens as well as through confirmation of on-target gene repression using perturb-seq, exhibiting 82%–92% median target knockdown. This genome-wide dual sgRNA library containing the top 2 on-target sgRNAs for 23,483 genes was subcloned into the library expression vector pU6-sgRNA Ef1alpha Puro-T2A-GFP derived from pJR85 (140095, Addgene) and modified to express a second sgRNA using the human U6 promoter as previously described (22). Knockdown efficiency of all guide sequences in this genome-wide sgRNA library was previously validated in K562 cells as part of a genome-wide Perturb-seq database, and these data are publicly available at https://gwps.wi.mit.edu/ A total of 1,137 nontargeting sgRNA pairs were also included as negative controls in the screen. To generate lentiviral pools, HEK293T cells were transfected with the sgRNA library along with packaging plasmids as described above, and viral supernatant was collected 72 hours after transfection. Lentiviral libraries were transduced into JH-2-002^dCas9-KRAB-BFP^ cells, cultured for 2 days following infection, selected in 1 μg/mL puromycin for 2 days, and then allowed to recover in 10% FBS in DMEM for 1 day. Cells were then sorted for GFP expression using FACS to obtain a pure population of 1 × 10^7^ cells, and cells were subsequently cultured to allow for 1 × 10^7^ cells per biological replicate. Two pellets of 1 × 10^7^ cells were subsequently frozen down at this T0 time point. The screen was subsequently carried out in biologic triplicate with 2 experimental groups, 1 receiving 2 daily doses of 2 Gy and 1 receiving no radiation. gDNA from all cells was harvested at the T14 (14-day) endpoint, and sgRNA barcodes were amplified and processed for sgRNA abundance library preparation using Q5 High-Fidelity DNA Polymerase (New England Biolabs [NEB]) and sequenced on an Illumina NovaSeq 6000 as previously described (57).

Enrichment or depletion of sgRNA abundances were determined by downsampling trimmed sequencing reads to equivalent amounts across all samples and then calculating the log_2_ ratio of sgRNA abundance in experimental conditions to the sgRNA abundance in control conditions at T14, or between sequencing reads from the T14 and T0 time points within the experimental or control conditions. Specifically, we computed normalized log_2_ ratios for radiation-treated sgRNA abundance at T14 compared with control T14 abundances to identify mediators of the radiation response and computed the normalized log_2_ ratios for untreated sgRNA abundance at T14 compared with T0 to identify regulators of cell fitness independent of treatment. Any sgRNAs not represented with at least 50 normalized sequencing reads across all replicates were excluded from analysis. Statistical significance was calculated using the Wald test comparing replicates across conditions without a log_2_ FC threshold. The screen was analyzed to identify significantly enriched or depleted guides with either vehicle treatment or radiation, with the latter being the focus for genetic mediators of the radiation response. Hits were prioritized by normalizing log_2_ ratios to the total number of population doublings in the screen. These phenotype log_2_ ratios were used for subsequent analysis and visualization. Genes were filtered at an adjusted *P* value of less than 0.05 for statistical significance. Significantly enriched gene ontologies were selected using ENRICHR (56) and visualized using the stringApp package in Cytoscape. Candidate genes for validation were selected from significantly enriched gene ontologies.

### Mouse subcutaneous allograft tumors.

Subcutaneous allografts were performed by implanting 5 million WT or sg*Ifnar1*-knockdown JW18.2 or JW23.3 MPNST allograft cells into the flanks of 5- to 6-week-old female C57/B6 WT mice (Charles River Laboratories) or into the flanks of 5- to 6-week-old female NU/NU mice (Harlan Sprague Dawley) housed in a 12-hour light/12-hour dark cycle at an average temperature of 73°F and 50% humidity. For radiation treatments, the desired radiation was delivered using the XRAD 320 irradiator (Precision X-ray), and custom 3D printed lead shielding was used to prevent radiation delivery to regions outside the palpable, visualized subcutaneous tumor. Tumors were measured 3 times per week using calipers.

### DNA methylation profiling.

Methylation profiling was performed using the Illumina EPIC array platform. Archival FFPE tissue blocks were screened, and DNA was extracted using the Qiagen FFPEasy kit, and 1,500 ng DNA was loaded per sample. DNA concentrations down to a minimum of 20 ng/μL (total: 1.5 μg) were considered acceptable for methylation array profiling. DNA quality was assessed by spectrophotometry, and clean-up was performed as needed using DNA precipitation. Preprocessing, normalization, and downstream analysis were performed in R using the minfi Bioconductor package, as previously reported (22, 58–60). Data were normalized via functional normalization and filtered according to the following criteria: (a) removal of probes targeting the X and Y chromosomes, (b) removal of probes containing a common SNP within the targeted CpG site or on an adjacent base pair, and (c) removal of probes not mapping uniquely to the human reference genome. Unsupervised hierarchical clustering (Euclidean distance, complete linkage method) was performed using the top 2,000 most variable probes as previously described (61). β Values were used for visualization of methylation levels [β = methylated/(methylated + unmethylated)], and M values were used for statistical analysis [M = log_2_(methylated/unmethylated)]. ConsensusClusterPlus (v.1.62.0) analysis (hierarchical clustering; k range of 2–10; 1,000 repetitions) was used to assess optimal cluster size and stability. CNVs were called using Conumee2 (62) with detailed inspection of methylation probes within selected chromosomal intervals in chromosome 9p21.3 to estimate gene-level deletions. Gene-level deletions were identified using both Conumee2’s segmentation and detail approaches. For the segmentation analysis, we considered any segment with a log_2_ segment mean of less than –1 and a *P* value of less than 0.01 overlapping MTAP, CDKN2A/B, or the IFN locus. For the detail analysis, significant genes were determined using *z* scores by comparing each gene’s log_2_-transformed copy number ratio against the distribution of bin values across the genome (log_2_ < –1, *z* score < –3, adjusted *P* < 0.05).

### scRNA-seq.

For mouse allograft scRNA-seq, tumors were minced with sterile razor blades and then enzymatically dissociated with papain (LS003, Worthington) at 37°C for 45 minutes. Samples were subsequently centrifuged for 5 minutes at 500*g*, resuspended in RBC lysis buffer (00-4300-54, eBioscience), incubated for 10 minutes at room temperature, and then resuspended in 5% FBS in PBS. Cell suspensions were serially filtered through 70 μm and 40 μm filters before being resuspended again in 5% FBS in PBS for manual cell counting using a hemacytometer. A total of 10,000 cells were loaded per scRNA-seq sample onto the 10X platform using the Chromium Single Cell 3′ Library and Gel Bead Kit v3.1 on a 10X Chromium controller (10X Genomics), per the manufacturer recommended default protocol and settings. Samples were sequenced on an Illumina NovaSeq at the UCSF Center for Advanced Technology, and the demultiplexed FASTQ files were processed using CellRanger for alignment to the mm10 reference genome, identification of empty droplets, and determination of a count threshold. Cellranger-generated filtered feature matrices were imported into a Seurat object. All downstream analyses were performed with Seurat v4.4 (63). For quality control, data were filtered on a per-sample basis to remove outliers in the gene count, the unique molecular identifier (UMI) count, mitochondrial genes, and ribosomal genes. The individual count matrices were normalized by SCTransform v2. Scanorama (https://github.com/brianhie/scanorama) (64) was used to perform data integration across datasets, and cluster number optimization was performed by comparing multiple cluster resolutions using Clustree, and the selected cluster resolution was examined by silhouette width analysis, which reported a mean width per cluster larger than 0. Genes differentially expressed in each cluster were identified using FindAllMarkers function (cutoff: min.pct = 0.25, logFC.threshold = 0.25, min.diff.pct = 0.1). The tumor versus nontumor cell microenvironment designation was based on *Xist* gene expression, which was expressed in female host mouse cells but not male MPNST tumor cells, scType automated cell identification (23), and manual inspection of differentially expressed gene markers as previously described (65).

### Statistics.

All experiments were performed as repeated, independent biologic replicates, and statistics were derived from the biologic replicates. The number of biologic replicates is indicated in each panel or figure legend. No statistical methods were used to predetermine sample sizes. Considering the rarity of MPNSTs and accounting for the number of genomic approaches used in this study, our total cohort size is similar to those of prior publications (22, 32, 33). The clinical samples used were retrospective and nonrandomized, and all samples were equally interrogated within the constraints of sufficient tissue for each analytical method. Cells and animals were randomized to experimental conditions, and no clinical, molecular, cellular, or animal data points were excluded from analysis. Unless otherwise specified, data are shown as the mean ± SEM. The statistical tests of choice were selected on the basis of the input data and are noted in the methods and figure legends. All statistical tests were 2 sided. Where appropriate, multiple hypothesis testing corrections were performed. Statistical significance thresholds are indicated in each figure legend and exact *P* values are provided whenever possible. A *P* value of less than 0.05 was considered significant.

### Study approval.

This study complied with all relevant ethics regulations and was approved by the UCSF IRB (13-12587, 17-22324, 17-23196, 18-24633, 22-37134) and the UCSF IACUC (AN200569-00). As part of routine clinical practice at UCSF, all patients included in this study signed an informed waiver of consent to contribute deidentified data to scientific research projects.

### Data availability.

Raw data are included in the Supporting Data Values supplemental file. Human tumor DNA methylation, bulk RNA-seq, and scRNA-seq data reported in this manuscript have been deposited in the NCBI Gene Expression Omnibus (GEO) (GEO records GSE290457, GSE290674, GSE289110, and GSE289111). The open-source software, tools, and packages used for data analysis in this study, as well as the version of each program, were as follows: ImageJ (v2.1.0), R (v3.5.3 and v3.6.1), cellranger (v6.1.2), Seurat R package (v4.4.0), Clustree (v0.5.0), Scanorama (v1.7.3), minfi (Bioconductor v3.10), ConsensusClusterPlus (Bioconductor v3.10), Heatmap.2 R package (gplots v3.13), and ggplot2 (v3.4.3). No custom software, tools, or packages were used. CRISPRi screen analysis code is available at GitHub (https://github.com/liujohn/CRISPRi-dual-sgRNA-screens/blob/main/module2/PhenotypeScores.R).

## Author contributions

All authors made substantial contributions to the conception or design of the study; the acquisition, analysis, or interpretation of data; or the drafting or revision of the manuscript. All authors approved the manuscript. All authors agree to be personally accountable for individual contributions and to ensure that questions related to the accuracy or integrity of any part of the work are appropriately investigated, resolved, and the resolution documented in the literature.

IZ designed, performed, and analyzed all experiments and bioinformatics analyses. GER and MDA performed methylation array copy number analysis and annotation. JC, K Miller, and JP performed qPCR, cell culture experiments including colony-forming assays, and mouse MPNST allograft experiments. K Mirchia assisted with assembling human tumor resection specimens, bioinformatics analysis, and pathologic review. SP performed scRNA-seq and assisted with bioinformatics analysis. VM, SEB, LJ, and RW provided key insights into the study design and provided clinical data. MP helped assemble tumor resection specimens, provided clinical data, performed pathologic review, and assisted with the study design. SJL provided critical experimental oversight including for the design of the CRISPR screens and assisted with bioinformatics analysis. HNV conceived, designed, and supervised the study.

## Funding support

This work is the result of NIH funding, in whole or in part, and is subject to the NIH Public Access Policy. Through acceptance of this federal funding, the NIH has been given a right to make the work publicly available in PubMed Central.

- HNV was supported by a Neurofibromatosis Therapeutic Acceleration Program Francis Collins Scholar Award.
- IZ was supported by a NIH T32 training grant (5T32GM141323-03) during the course of this work.
- This study was supported in part by the HDFCCC Laboratory for Cell Analysis Shared Resource Facility through a grant from the NIH (P30CA082103).

## Supplementary Material

Supplemental data

Supplemental tables 1-6

Supporting data values

## Version 1 03/02/2026

Electronic publication
