# Supplementary material for: IFN signaling is associated with radiotherapy response in malignant peripheral nerve sheath tumors
Source: J Clin Invest. 2026 Mar 2;136(5):e195652. doi: 10.1172/JCI195652 (PMC12948414; doi:10.1172/JCI195652)
Supplement: Supplemental data [file jci-136-195652-s020.pdf]

## Supplementary Information

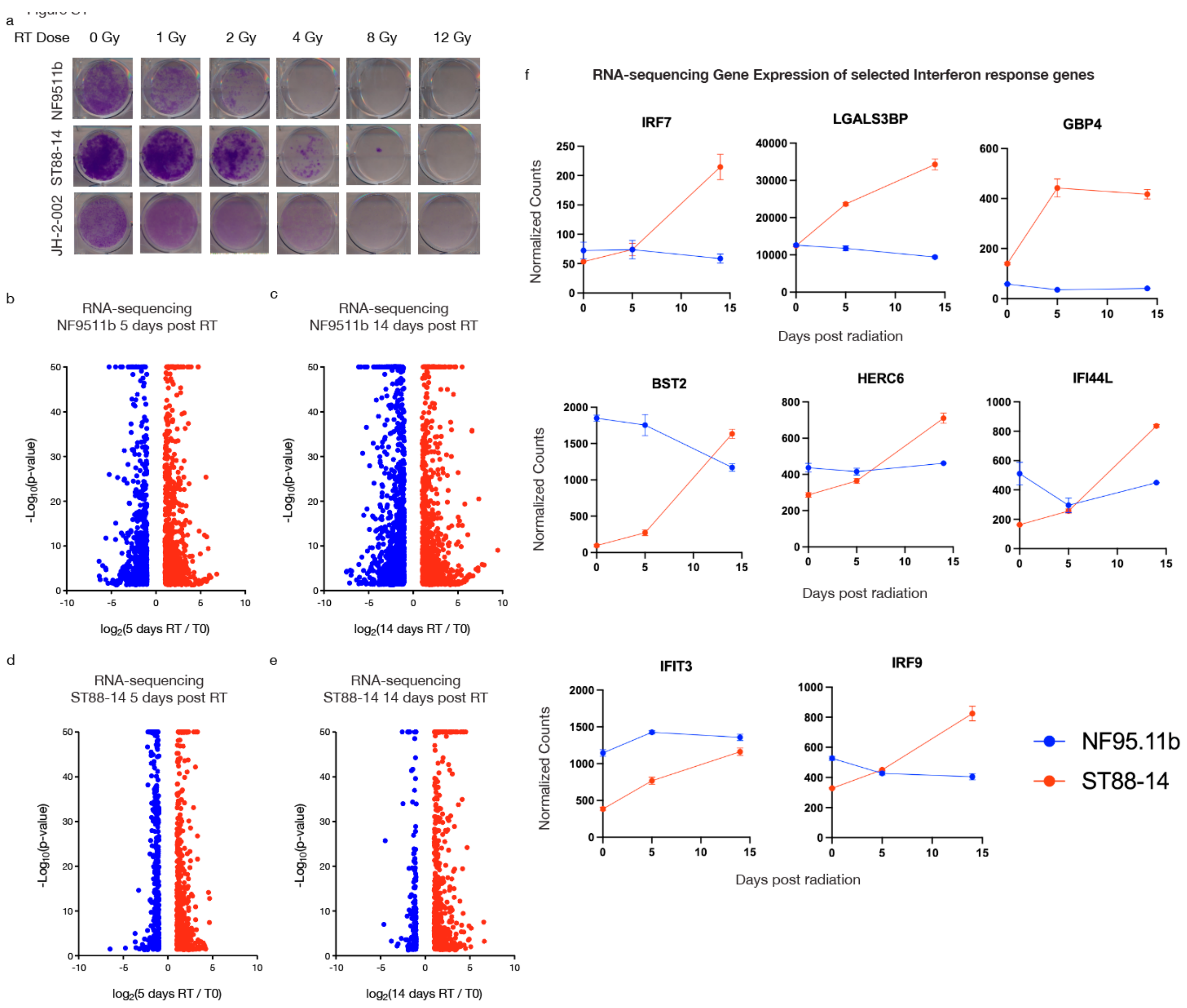


**Supplementary Figure 1. Bulk RNA-sequencing analysis of NF9511b pNF or ST88-14 MPNST cells at early (5 days) or late (14 days) timepoints following radiation.** a. Radiation dose response from 0-12 Gray (Gy) followed by colony forming assays revealed MPNST cells showed increased viability compared to pNF cells. b. Volcano plots depicting significantly modulated genes in NF9511b pNF cells at b. 5 days (793 upregulated genes, 757 downregulated genes) or c. 14 days (937 upregulated genes, 1178 downregulated genes) following radiation and in ST88-14 MPNST cells at d. 5 days (651 upregulated genes, 652 downregulated genes) or e. 14 days (605 upregulated genes, 186 downregulated genes) following radiation. Genes were considered significant at $p < 0.05$ and -log10(p-value) scores were capped at 50 for visualization purposes. f. ST88-14 MPNST cells, but not NF9511.b pNF cells, significantly upregulated IFN signaling genes at both early and late RT response timepoints and downregulated mitosis-associated genes.

Figure S2

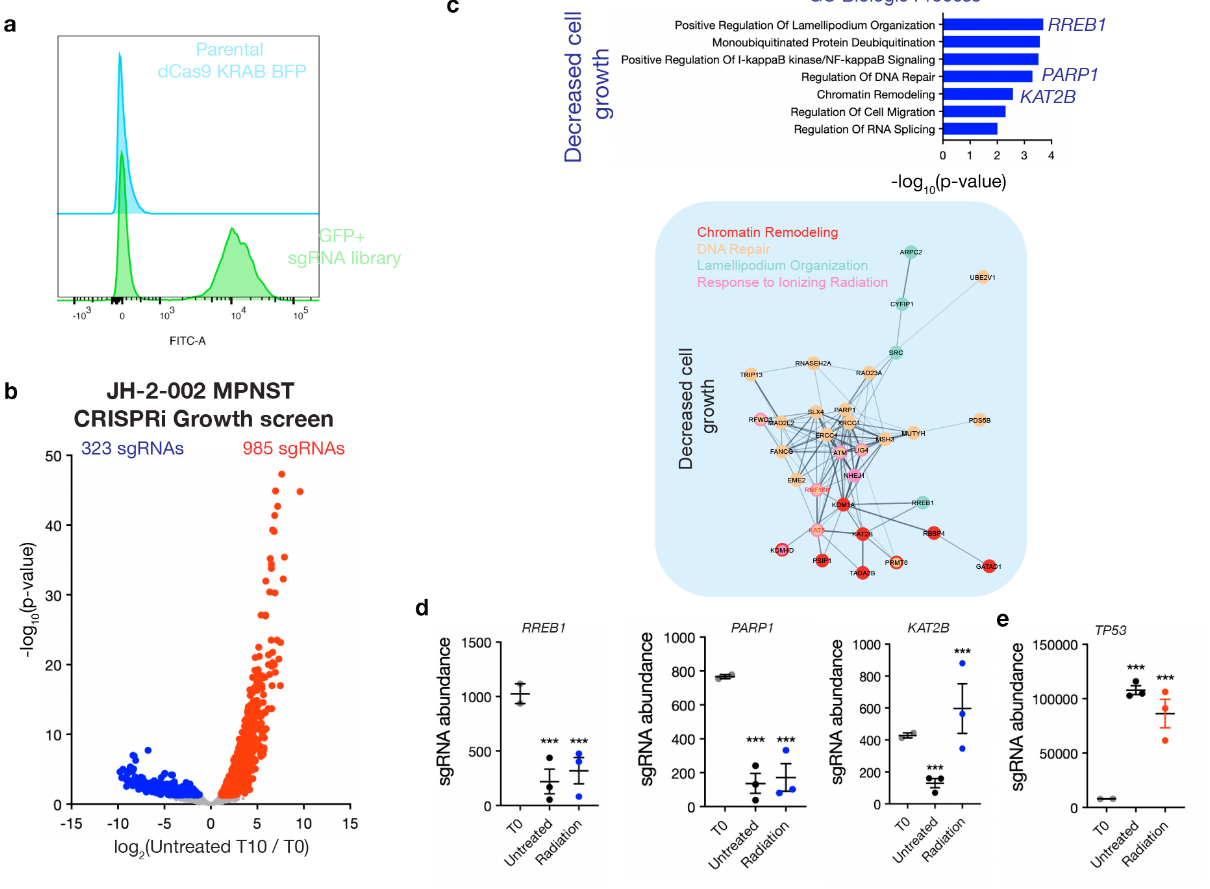


**Supplementary Figure 2. Genome wide CRISPRi growth screen (T14 untreated compared to T0) analysis in JH-2-002 MPNST cells.** a. Flow cytometry demonstrating gating parameters for GFP+ sgRNA library (bottom) compared to negative control, un-transduced dCas9 KRAB BFP parental cell line (top). b. Volcano plot depicting 323 significantly depleted sgRNAs (blue) and 985 significantly enriched sgRNAs (red) mediating MPNST cell growth at T14 compared to initial T0 timepoint. c. Gene ontology analysis of genes required for MPNST cell growth reveals genes and processes known to be important in MPNSTs including d. Ras target transcription factors (*RREB1*), DNA repair (*PARP1*), or chromatin remodeling (*KAT2B*). e. In addition, sg*TP53* was amongst the top hits mediating increased MPNST cell growth, serving as a positive control. *** $p<0.01$ compared to T0, Wald test.

Figure S3

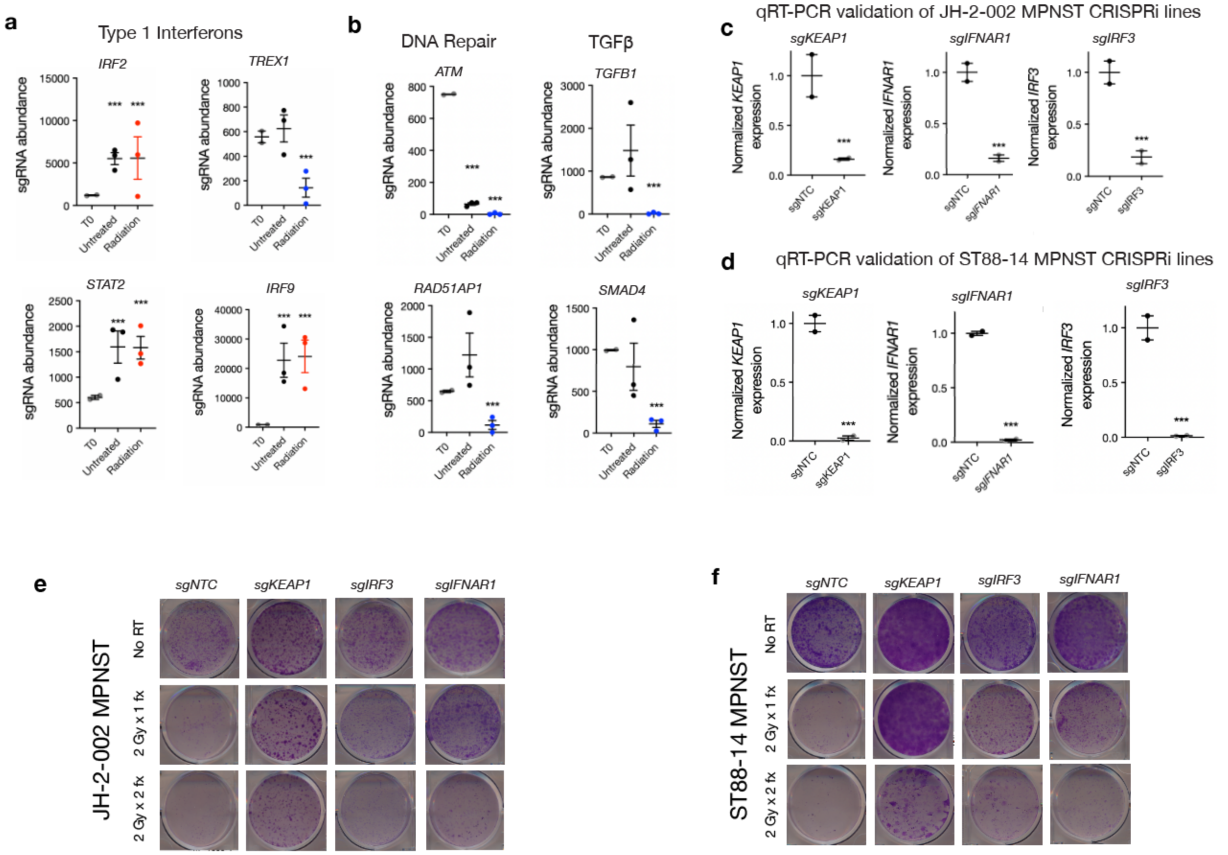


**Supplementary Figure 3. Selected CRISPRi screen hits significantly modulate radiation response.** a. Multiple components of type 1 interferon signaling (*IRF2, STAT2, IRF9)* mediate radiation resistance while repression of the negative regulator of STING *TREX1* significantly mediates radiation sensitivity. b. Consistent with their well-established roles in cell autonomous radiation response, DNA repair (*ATM, RAD51AP1)* and TGFβ signaling (*TGFB1, SMAD4*) mediate radiosensitivity. c. qRT-PCR validation in JH-2-002 or d. ST88-14 MPNST cells of selected JH-2-002 CRISPRi screen hits (*sgKEAP1, sgIRF3, sgTMEM173, sgSTAT1)*. Colony forming assays validates relative radioresistance of selected CRISPRi screen hits in e. JH-2-002 and f. ST88-14 MPNST cells relative to sgNTC control cells. * $p<0.05$, *** $p<0.01$ compared to T0, Wald test.

Figure S4

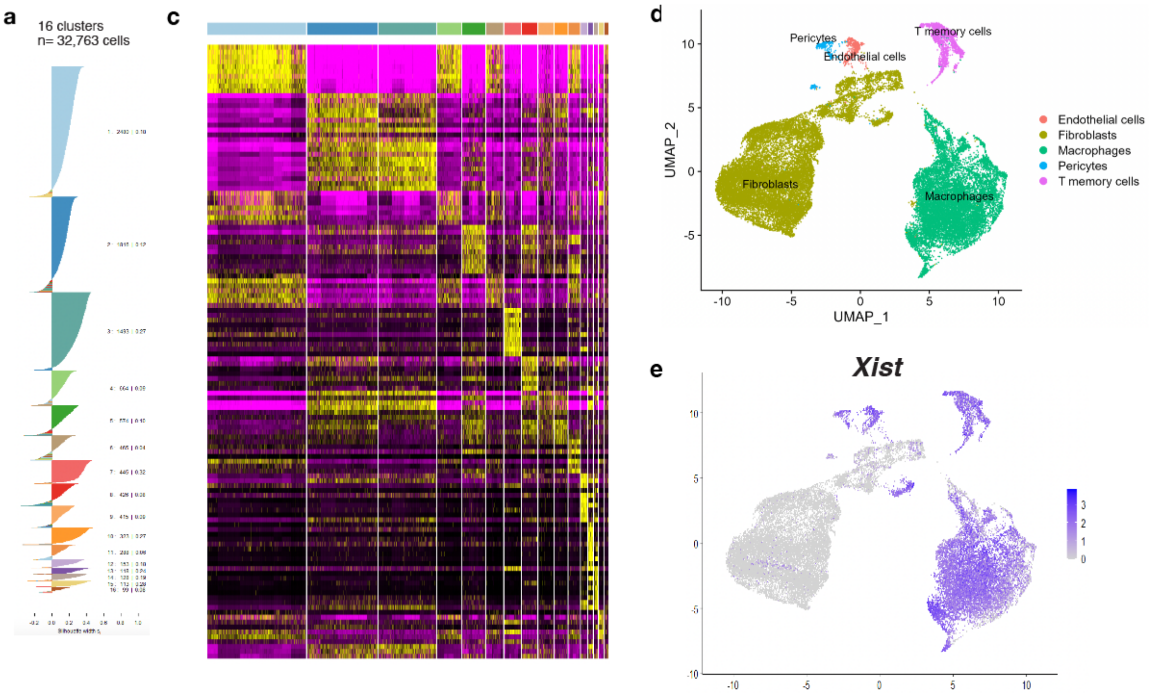


b

| | 0 | 1 | 2 | 3 | 4 | 5 | 6 | 7 | 8 | 9 | 10 | 11 | 12 | 13 | 14 | 15 |
|---|---|---|---|---|---|---|---|---|---|---|---|---|---|---|---|---|
| Untreated 1 | 874 | 1150 | 1138 | 13 | 543 | 516 | 145 | 9 | 432 | 93 | 99 | 54 | 31 | 37 | 38 | 74 |
| Untreated 2 | 1895 | 1292 | 1071 | 584 | 289 | 25 | 276 | 68 | 73 | 293 | 281 | 178 | 111 | 113 | 47 | 82 |
| Untreated 3 | 1632 | 1258 | 430 | 774 | 217 | 72 | 194 | 699 | 47 | 365 | 205 | 112 | 68 | 52 | 83 | 65 |
| Radiation 1 | 1223 | 1367 | 832 | 580 | 268 | 54 | 260 | 426 | 488 | 192 | 95 | 88 | 78 | 55 | 62 | 67 |
| Radiation 2 | 708 | 361 | 387 | 18 | 120 | 226 | 123 | 20 | 130 | 23 | 73 | 17 | 39 | 41 | 29 | 19 |
| Radiation 3 | 742 | 458 | 580 | 24 | 293 | 307 | 181 | 72 | 94 | 69 | 103 | 39 | 35 | 35 | 38 | 26 |
| Radiation 4 | 1295 | 101 | 434 | 84 | 204 | 230 | 232 | 44 | 12 | 65 | 118 | 37 | 26 | 41 | 63 | 15 |

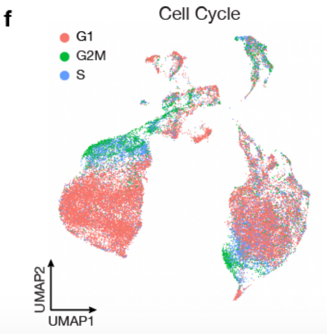


**Supplementary Figure 4. Single cell RNA-sequencing (scRNA-seq) of irradiated and control mouse MPNST allograft tumors implanted subcutaneously in C57/B6 mice.** a. Silhouette analysis reveals 16 clusters across 32,763 cells across b. 3 untreated and 4 irradiated tumors. c. Heatmap for cluster marker genes across all clusters integrated with d. automated cell type assignment (SCtype), e. *Xist* expression to define female host mouse microenvironment cells, and f. cell cycle expression signature leads to robust cell type classification.

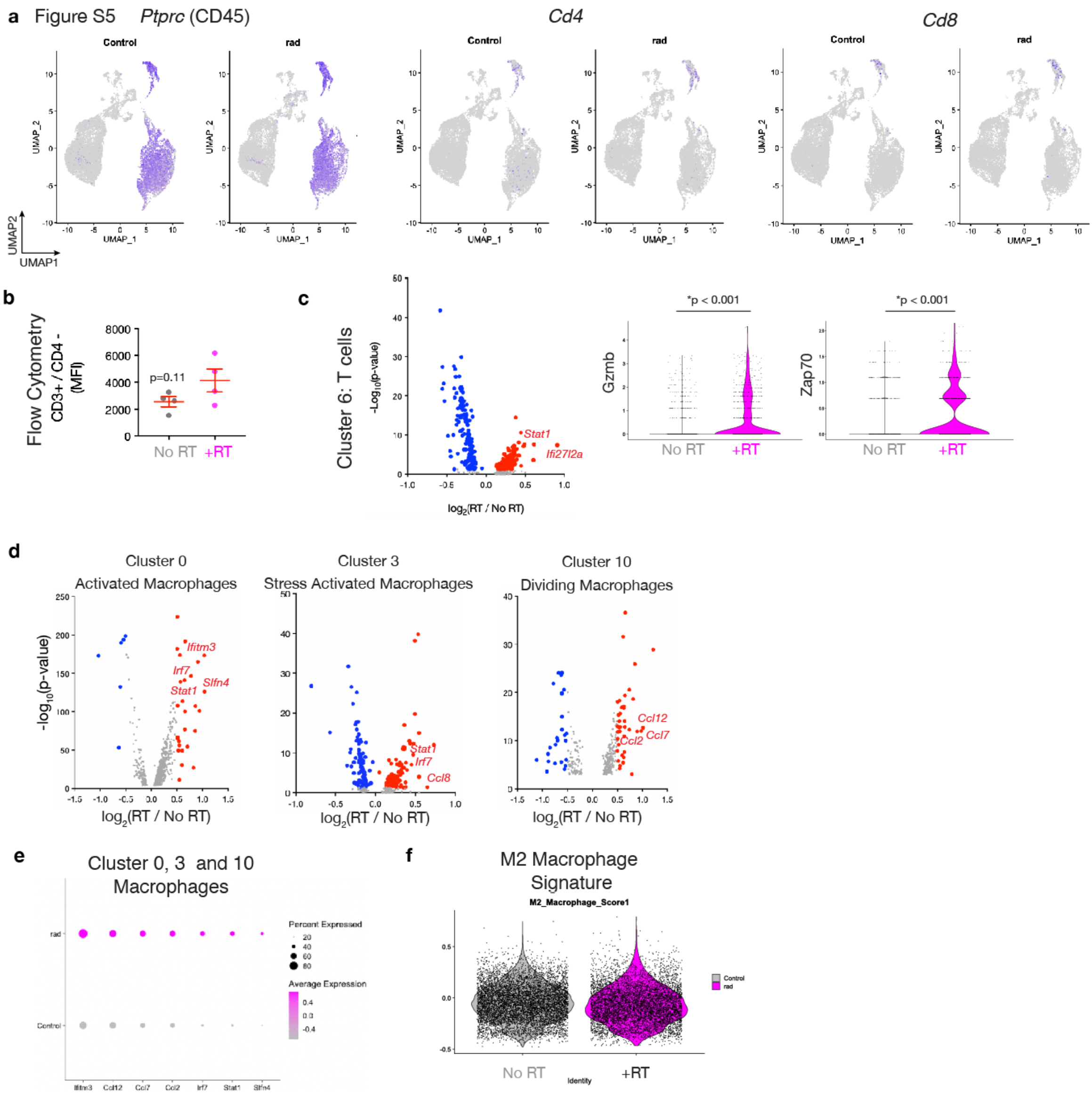


**Supplementary Figure 5. Differential expression analysis of non-tumor T/NK cells (Cluster 6) and macrophages (Clusters 0, 3, and 10).** a. *Ptprc* (CD45) expression as a pan leukocyte marker identifies all cells of the leukocyte lineage while CD4/CD8 is restricted to Cluster 6. b. Flow cytometry reveals a trend toward increased CD3+/CD4- (reflecting the CD8+ compartment) following radiation therapy (RT) compared to control (no RT) cells. c. Differential gene expression analysis between irradiated (RT) and control (no RT) Cluster 6 T/NK cells reveals induction of T cell activation (*Gzmb, Zap70*) and interferon gene circuits (*Ifi27l2a, Stat1*) following radiation. d,e. Differential gene expression analysis between irradiation (RT) and control (no RT) macrophage clusters reveals induction of interferon gene circuits (*Irf7, Ifitm3, Stat1)* and multiple chemokines (*Ccl2, Ccl7, Ccl8, Ccl12*) following radiation. f. Irradiation does not significantly alter M2 macrophage polarization.

Figure S6

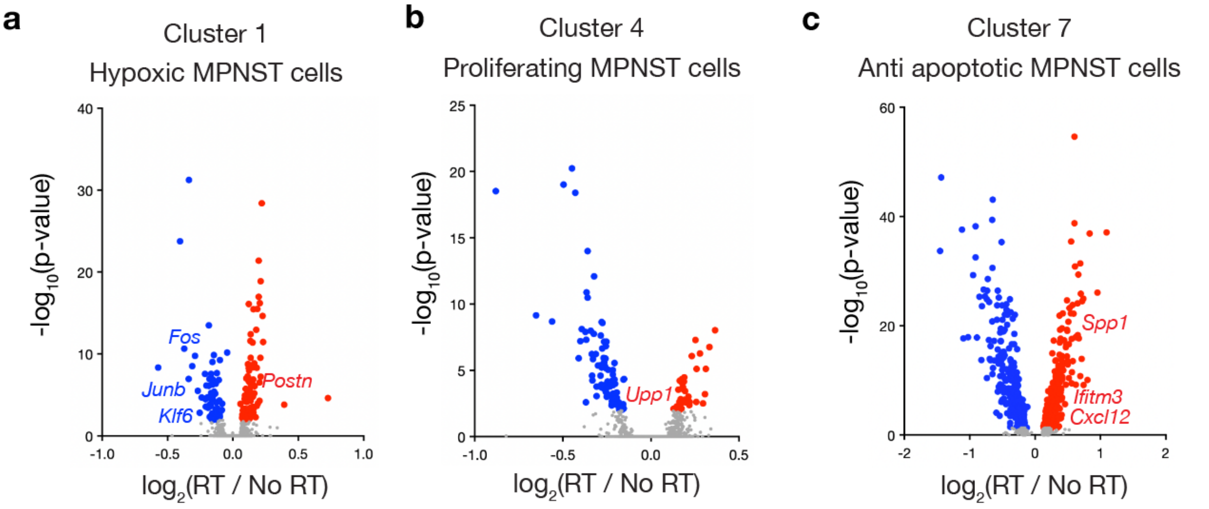


**Supplementary Figure 6.** Differential expression analysis of a. hypoxic MPNST cells (Cluster 1), b. proliferating MPNST cells (Cluster 4), or c. anti-apoptotic MPNST cells (Cluster 7) reveals induction of interferon signaling *(Spp1, Ifitm3*) and chemokine secretion (*Cxcl12*) in Cluster 7 cells following irradiation.

Figure S7

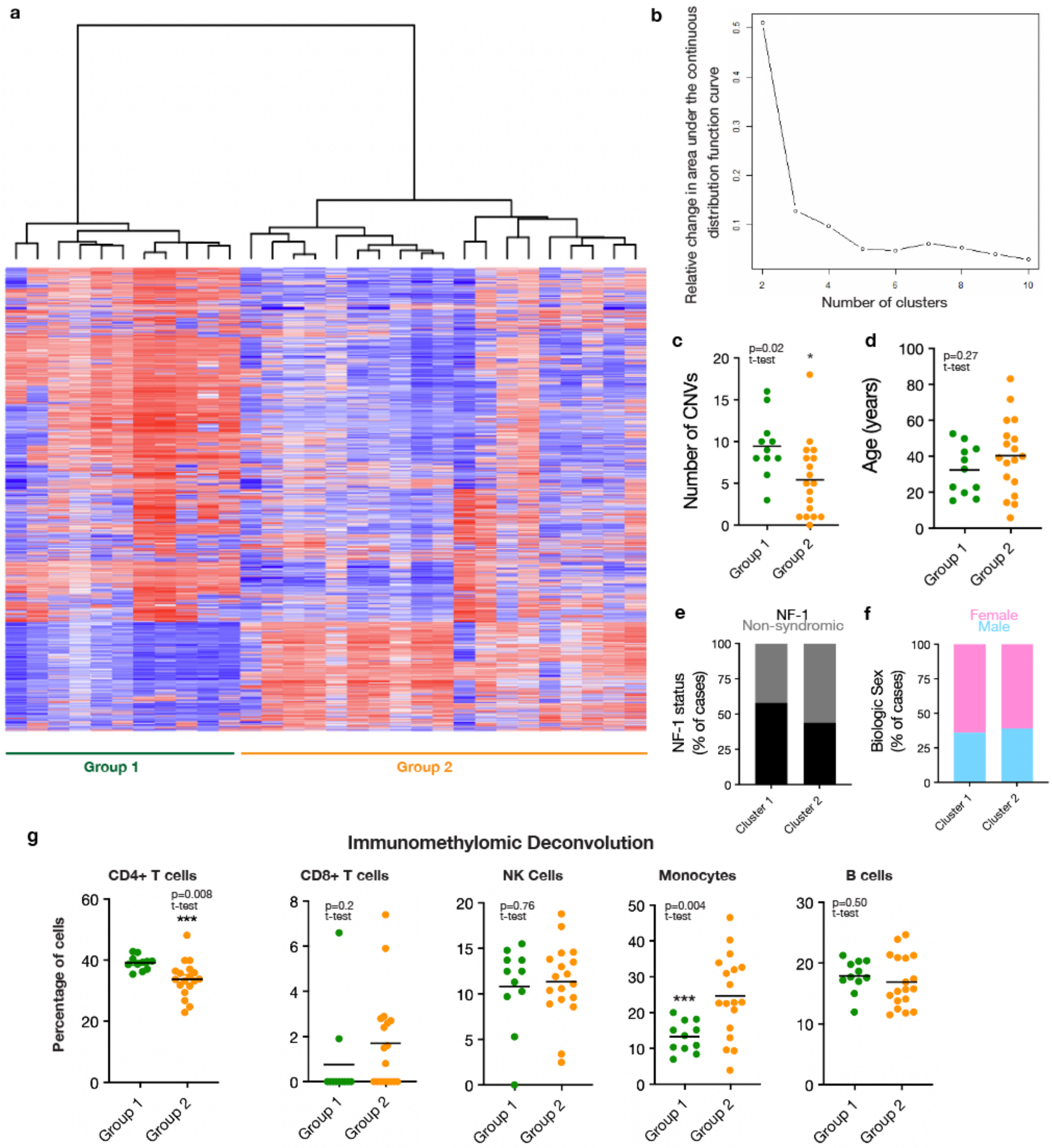


**Supplementary Figure 7. Methylation array analysis of human patient MPNST resection specimens treated with postoperative radiation therapy.** a. Unsupervised hierarchical clustering heatmap of top 2,000 most variable probes identifies b. two epigenetic groups based on the change in area under the curve for consensus cumulative distribution function (CDF), which shows minimal appreciable increase for greater than 2 clusters. c. Group 2 tumors have significantly increased CNVs but no significant difference in d. age, e. NF-1 status, or f. biologic sex. g. Immunomethylomic deconvolution of different microenvironmental cell types revealed

Group 1 tumors had significantly increased CD4+ T-cells, significantly decreased monocytes, and a trend toward decreased CD8+ T-cells.

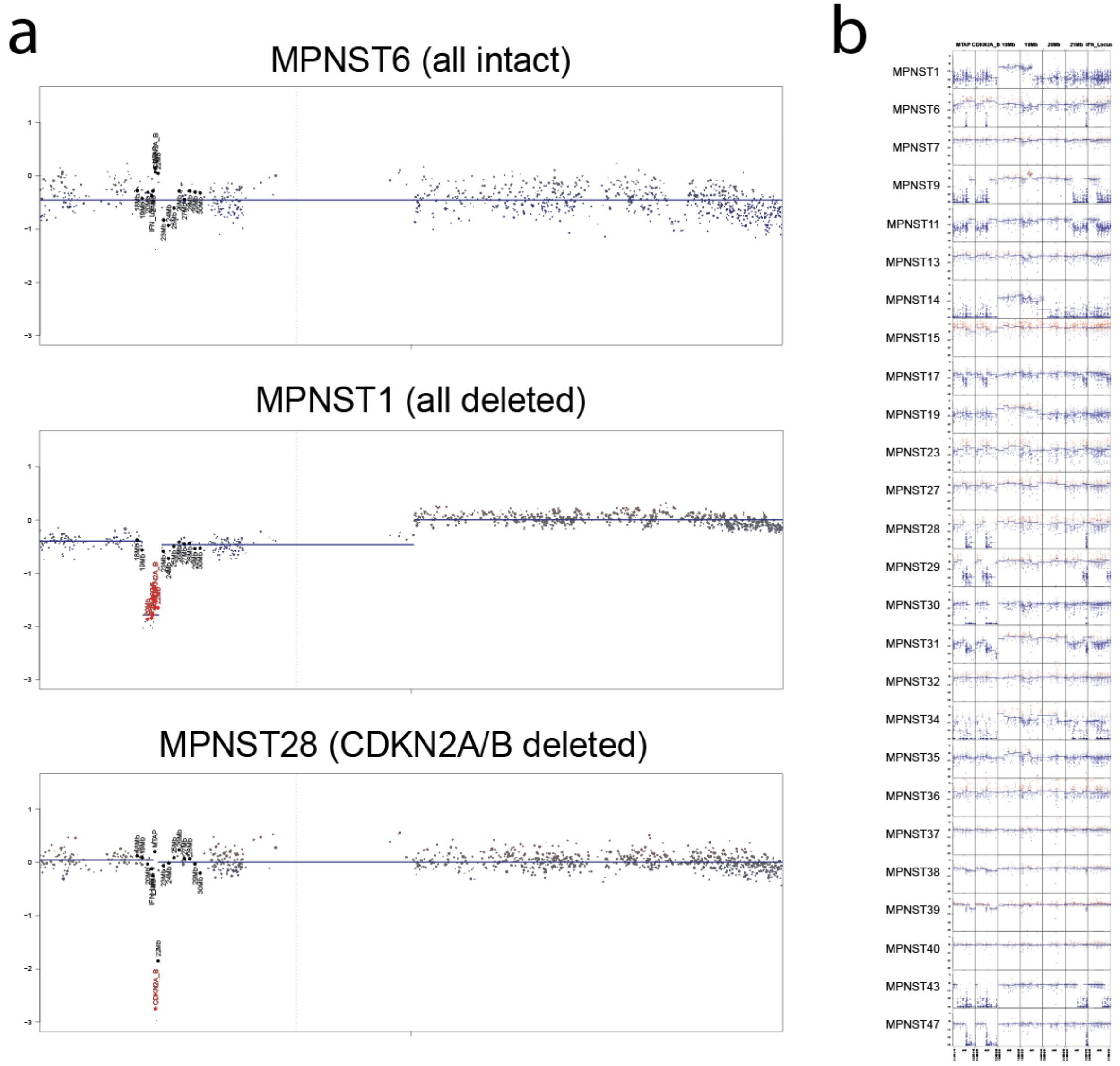


**Supplementary Figure 8. DNA methylation array based copy number variant (CNV) estimation.** a. Example CNV segmentation plots for a chromosome 9p intact sample (MPNST6), a sample with deletion of all three genes of interest *CDKN2A/B, MTAP,* and the *IFN* locus (MPNST1), and a sample with *CDKN2A/B* deletion alone (MPNST28). b. Gene level segmentation plots for *MTAP, CDKN2A/B,* and the *IFN* locus for all MPNSTs.

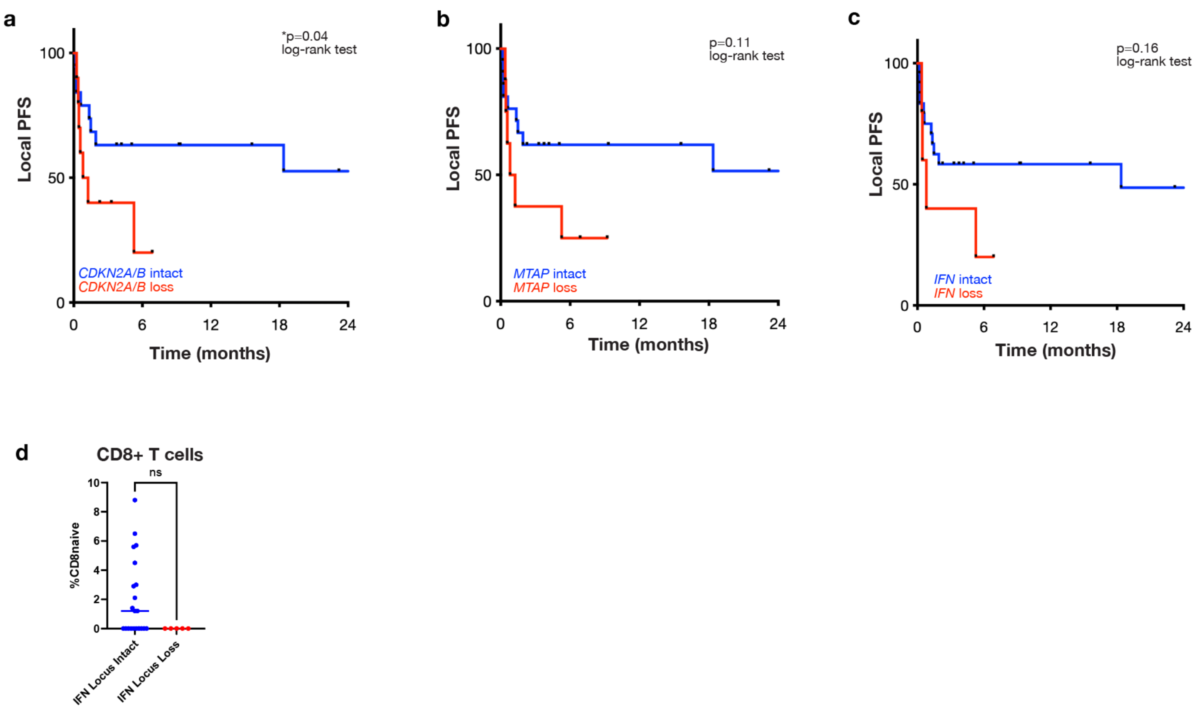


**Supplementary Figure 9. Analysis of specific genes included in the chromosome 9p deletion reveals correlations with local control.** a. *CDKN2A/B* loss is associated with significantly worse local progression free survival (PFS) while b. *MTAP* loss or c. *IFN* locus deletion show non-significant trends toward poorer PFS. d. Tumors from patients harboring an *IFN* locus deletion demonstrate a decrease in CD8 naïve T cells (p=0.08).

**Supplementary Tables.**

**Supplementary Table 1.** Normalized gene expression values for bulk RNA-sequencing of irradiated plexiform neurofibroma NF9511b or MPNST ST88-14 cells.

**Supplementary Table 2.** Analysis of MPNST JH002-2 CRISPRi screen comparing sgRNA enrichment between cells treated with the radiation (2 Gy x 2 fractions) or no treatment for 10 days

**Supplementary Table 3.** Gene Ontology analysis for CRISPRi screen results

**Supplementary Table 4.** Cluster marker gene list for scRNA-sequencing of tumor cells from irradiated 2 Gy x 5 fractions (n=4) or untreated (n=3) JW23.3 MPNST subcutaneous allografts.

**Supplementary Table 5.** Gene Ontology analysis for single cell RNA-sequencing cluster marker genes.

**Supplementary Table 6.** Methylation array based CNV calling from MPNST samples.
